# Clinical characteristics, tumor‐infiltrating lymphocytes, and prognosis in HER2‐low breast cancer: A comparison study with HER2‐zero and HER2‐positive disease

**DOI:** 10.1002/cam4.6290

**Published:** 2023-06-27

**Authors:** Yujie Lu, Yiwei Tong, Xiaochun Fei, Xiaosong Chen, Kunwei Shen

**Affiliations:** ^1^ Department of General Surgery, Comprehensive Breast Health Center, Ruijin Hospital Shanghai Jiao Tong University School of Medicine Shanghai China; ^2^ Department of Pathology, Comprehensive Breast Health Center, Ruijin Hospital Shanghai Jiao Tong University School of Medicine Shanghai China

**Keywords:** breast cancer, HER2‐low, sTILs, survival

## Abstract

**Introduction:**

HER2‐low breast cancer is a gradually recognized and unexplored group of diseases. We aimed to investigate the clinical and prognosis features and to identify the role of stromal tumor‐infiltrating lymphocytes (sTILs) in this population.

**Methods:**

Consecutive primary breast cancer patients treated between January 2009 to June 2013 were retrospectively reviewed. HER2‐low was defined as immunohistochemistry (IHC) 1+, or 2+ and fluorescence in situ hybridization (FISH) negative. sTILs were scored following the international guidelines. Clinicopathologic features and survival were compared according to HER2 and sTILs category.

**Results:**

A total of 973 breast cancer patients were enrolled, including 615 (63.2%) HER2‐low patients. HER2‐low patients shared more similarity with HER2‐0 cases in clinicopathological features. sTILs in HER2‐Low patients was comparable to HER2‐0 patients (*p* = 0.064), both significantly lower than HER2‐positive ones (*p* < 0.001). Meanwhile, tumors with sTILs ≥50% accounted for the least proportion of HER2‐low cases (*p* < 0.001). HER2 status had no significant influence on recurrence‐free survival (RFS, *p* = 0.901) in the whole population. However, in the estrogen receptor (ER)‐negative subgroup, HER2‐low was related to worse RFS (*p* = 0.009) and OS (*p* = 0.001) compared with HER2‐positive ones. sTILs increment was an independent favorable prognostic factor in the whole (OS, *p* = 0.003; RFS, *p* = 0.005) and HER2‐low population (OS, *p* = 0.007; RFS, *p* = 0.009) after adjusted to clinicopathological parameters.

**Conclusions:**

HER2‐low patients shared similar clinicopathological features with HER2‐0 rather than HER2‐positive cases and had relatively low sTILs. ER‐negative/HER2‐low patients had significantly inferior survival. sTILs increment was independently associated with favorable survival in the HER2‐low group, suggesting a potential benefit from a novel treatment strategy.

## 1 INTRODUCTION

Breast cancer is the most commonly diagnosed malignancy worldwide.^1^ Human epidermal growth factor receptor‐2 (HER2) gene amplification and subsequent HER2 protein overexpression is identified in approximately 15% of invasive breast cancer cases and is associated with poor prognosis.^2^ Meanwhile, HER2 remains a well‐established therapeutic target, which predicts substantial clinical benefit from anti‐HER2 agents including trastuzumab.^3^, ^4^ In clinical practice, HER2 status is assessed by immunohistochemistry (IHC) and/or fluorescence in situ hybridization (FISH), according to American Society of Clinical Oncology/College of American Pathologists (ASCO/CAP) guidelines.^5^ In brief, patients with HER2 IHC 3+ or 2+ in combination with positive FISH result (FISH+) are considered HER2‐positive and eligible for anti‐HER2‐targeted therapy,^6^ whereas the others are defined as HER2‐negative. As researches move along, we come to recognize that HER2 expression is rather continuous than binominal.^7^ Apart from those who do not express any HER2 protein (HER2‐0), a large proportion of tumors do show a low to moderate expression of HER2 protein by IHC, but without HER2 gene amplification. Clinical features, treatment, and outcomes for these so‐called HER2‐low tumors (HER2 IHC 1+ or 2+ and FISH negative) have become new fields of interest.

HER2‐low breast cancers make up 40%–60% of all breast cancer cases.^8^, ^9^, ^10^ Currently, the standard of care in these cases is generally based on hormone receptor (HoR) status, which is hormone therapy for HoR+ patients and chemotherapy for triple‐negative (TN) patients, according to clinical guidelines.^11^ Although trastuzumab showed convincing benefits for HER2‐positive patients,^6^, ^12^ the application of trastuzumab did not bring survival improvement in HER2‐low patients, as shown in the randomized phase 3 NSABP‐B47 trial.^13^ Over the past decade, the rapid development of novel HER2‐targeted antibody‐drug conjugates (ADCs) has shed light on new treatment option in the HER2‐low population. Recently released first report of DESTINY‐Breast04 has brought us exciting positive results that trastuzumab‐deruxtecan (DS‐8201) could almost double the progression‐free survival in advanced HER2‐low breast cancer patients compared with regular treatment.^14^ Meanwhile, trastuzumab‐duocarmazine (SYD985) has also exhibited notable therapeutic activity in HER2‐low breast cancer patients with an objective response rate of 31.9% (ClinicalTrials.gov identifier: NCT02277717).^15^ With these delightful results, the management of HER2‐low breast cancers has ushered into a new era, demanding a more thorough understanding of the molecular features of this new entity of disease.

Over the past decade, the role of stromal tumor‐infiltrating lymphocytes (sTILs) in tumor microenvironments has been well recognized. sTILs are more abundant in HoR‐negative tumors rather than HoR‐positive cases and show different prognostic and predictive values.^16^, ^17^, ^18^, ^19^, ^20^ For instance, in HER2‐positive breast cancer patients, where antibody‐dependent cell‐mediated cytotoxicity (ADCC) and phagocytosis (ADCP) are the major mechanisms of action of anti‐HER2 treatment,^21^, ^22^ sTILs infiltration was regarded as a strong predictive and prognostic factor in both early^16^, ^17^, ^19^, ^23^, ^24^ and late setting.^25^ In addition to HER2‐positive cases, high sTILs infiltration is also shown to be strongly associated with superior survival in TN, but unfavorable prognosis in HoR‐positive patients.^26^ Nevertheless, no study so far has explored the role of sTILs in the HER2‐low population. As a result, a better understanding of not only the clinicopathologic characteristics but also the immune features of HER2‐low tumors is essential.

To this end, we first aim to compare the clinicopathologic features in breast cancer patients with different HER2 statuses. Second, to investigate the correlation between sTILs and HER2 status as well as to identify the impact factors for sTILs in HER2‐low patients. Moreover, we intend to establish the prognostic value of factors including sTILs in the HER2‐low population.

## 2 METHODS

### 2.1 Study population

Consecutive breast cancer patients receiving surgical treatment at Comprehensive Breast Health Center, Ruijin Hospital between January 2009 and December 2013 were retrospectively reviewed. The inclusion criteria were (a) histologically proven invasive breast cancer; (b) female gender; (c) unifocal disease; (d) complete IHC, FISH, and sTILs results; and (e) complete follow‐up information. Those with de novo stage IV disease were excluded (Figure S1). The informed consent of this study was waived for this retrospective study only including anonymous data in the existing database, which was approved by the independent Ethical Committees of Ruijin Hospital, Shanghai Jiao Tong University School of Medicine. (Approval code: 2020‐309, date of approval: September 17, 2020).

### 2.2 Histo‐pathologic evaluation and HER2 testing algorithms

Tumor histo‐pathologic examination was accomplished in the Department of Pathology, Ruijin Hospital, independently by at least two experienced pathologists. The criteria adopted for estrogen receptor (ER), progesterone receptor (PR), and Ki67 IHC evaluation were according to the latest ASCO/CAP guidelines,^5^ as described in our previous studies.^27^, ^28^

The assessment of HER2 status took place in the context of normal clinical care, where at least two pathologists independently identified HER2 status according to the latest ASCO/CAP guidelines. The algorithms for HER2 testing were to first test HER2 expression by IHC and to carry out a subsequent FISH test using *HER2/CEP17* dual probe in case of IHC 2+, intratumoral heterogeneity, or unsolved discordance between observers.^5^ HER2 test result was reported as positive if IHC 3+, or IHC 2+ and FISH positive (dual‐probe *HER2/CEP17* ratio of ≥2.0 with an average *HER2* copy number ≥4.0 signals/cell, or dual‐probe *HER2/CEP17* ratio of <2.0 with an average *HER2* copy number ≥6.0 signals/cell).^9^ HER2‐low was defined as IHC 1+, or IHC2+ and FISH negative (dual‐probe *HER2/CEP17* ratio of <2.0 with an average *HER2* copy number <6.0 signals/cell, or dual‐probe *HER2/CEP17* ratio of ≥2.0 with an average *HER2* copy number <4.0 signals/cell).^9^ HER2‐0 is referred to HER2 IHC 0.

Stromal immune infiltrate was noted as the percentage of stromal immune cells infiltration within tumor borders, which were collected based on our previous studies^29^, ^30^, ^31^ and several unpublished researches, which were retrospectively re‐read on hematoxylin–eosin staining sections from the formalin‐fixed paraffin‐embedded (FFPE) samples according to the International TIL Working Group Guidelines.^32^ Pretreatment tumor samples of core needle biopsy were used for IHC and sTILs assessment in the patients receiving neoadjuvant chemotherapy (NAC). In the case of heterogeneity, hot‐spot and cold‐spot regions were spared and the average value was recorded. Patients were then categorized into high infiltrate (sTILs ≥ 50%), intermediate infiltrate (10% ≤ sTILs < 50%), and low infiltrate (sTILs < sTILs<10%) groups.

### 2.3 Data collection

Clinicopathologic data of the included patients were retrospectively retrieved from Shanghai Jiao Tong University Breast Cancer Database (SJTU‐BCDB). Cancer history referred to the history of nonbreast malignancy before the diagnosis of breast cancer. Follow‐up was accomplished by specialized breast cancer nurses in our center. Patient prognosis was calculated according to the STEEP criteria, in terms of recurrence‐free survival (RFS), which was calculated from the date of surgery to the recurrence of the tumor, including ipsilateral, local/regional, or distant recurrence, and death from any cause.^33^ The secondary endpoint was overall survival (OS), which was calculated from the date of surgery till death from any cause.^33^ The last follow‐up was completed by June 2021.

### 2.4 Statistical analysis

The clinicopathologic features were compared according to HER2 status by univariate Chi‐square test and multinomial logistic regression reporting odds ratio (OR) with 95% confidence interval (CI). One‐way ANOVA was adopted to compare the distribution of sTILs by different HER2 statuses. Impact factors for sTILs in HER2‐low population were identified through univariate and multivariate logistic regression. Censoring time (T‐CENS) method to calculate median follow‐up. Kaplan–Meier curves were conducted to compare clinical outcomes according to HER2 status and sTILs infiltration. Stratified Mantel–Haenszel test was then applied to estimate the hazard ratio (HR) with 95% CI in subgroup analysis. Statistical analysis and image construction were performed using IBM SPSS version 25 (SPSS, Inc.) and GraphPad Prism version 8.0 (GraphPad Software), and a two‐sided *p* value of <0.05 was considered statistically significant.

## 3 RESULTS

### 3.1 Baseline characteristics by HER2 status

Overall, data from 973 early breast cancer patients were finally analyzed, among whom 126 were HER2‐0, 615 were HER2‐low, and 232 were HER2‐positive (Figure S1). Baseline clinicopathologic features and treatment information are shown in Table 1. Among the included population, the median age was 55 (range 23–92) years. Thirty of 167 patients undergoing NAC achieved pCR. In the rest 943 patients with surgical resection tumor samples, invasive ductal carcinoma (IDC) was diagnosed in 90.0% of patients, and 36.5% had grade III tumors. Node‐positive disease was found in 29.2% of patients.

**TABLE 1:** Baseline characteristics according to HER2 status.

Characteristics	HER2‐0 *N* = 126 (%)	HER2‐low *N* = 615 (%)	HER2+ *N* = 232 (%)	*p* value
Age, years (median, range)	53.0 (27–86)	56.0 (24–92)	53.0 (23–90)	<0.001
Age, years				
<55	69 (54.8)	285 (46.3)	131 (56.5)	0.016
≥55	57 (45.2)	330 (54.7)	101 (43.5)	
Menstruation				
Pre/perimenopausal	55 (43.7)	233 (37.9)	102 (44.0)	0.186
Postmenopausal	71 (56.3)	382 (62.1)	130 (56.0)	
Prior cancer history				
Yes	5 (4.0)	25 (4.1)	7 (3.0)	0.778
No	121 (96.0)	590 (95.9)	224 (97.0)	
CCI				
0–1	87 (69.0)	418 (68.0)	177 (76.3)	0.059
≥2	39 (31.0)	197 (32.0)	55 (23.7)	
Histology^a^				
IDC	102 (85.0)	529 (88.2)	218 (97.8)	<0.001
Non‐IDC	18 (15.0)	71 (11.8)	5 (2.2)	
Grade^a^				
I–II	50 (41.7)	355 (59.2)	90 (40.4)	<0.001
III	45 (37.5)	172 (28.7)	127 (57.0)	
Unknown	25 (20.8)	73 (12.1)	6 (2.6)	
Tumor size, cm^a^				
≤2	75 (62.5)	369 (61.5)	101 (45.3)	<0.001
>2	45 (37.5)	231 (38.5)	122 (54.7)	
Lymph node status^a^				
Negative	99 (82.5)	448 (74.7)	121 (54.3)	<0.001
Positive	21 (17.5)	152 (25.3)	102 (45.7)	
LVI^a^				
Yes	5 (4.2)	18 (3.0)	7 (3.1)	0.801
No	115 (95.8)	582 (97.0)	216 (96.9)	
ER status				
Positive	100 (79.4)	535 (87.0)	109 (47.0)	<0.001
Negative	26 (20.6)	80 (13.0)	123 (53.0)	
PR status				
Positive	85 (67.5)	461 (75.0)	79 (34.1)	<0.001
Negative	41 (32.5)	154 (25.0)	153 (65.9)	
Ki67				
<14%	60 (47.6)	244 (39.7)	45 (19.4)	<0.001
≥14%	66 (52.4)	371 (60.3)	187 (80.6)	
sTILs (mean, range)	14.3% (0–70)	10.7% (0–80)	19.4% (0–80)	<0.001
sTILs categories				
≥50%	12 (9.5)	33 (5.4)	34 (14.7)	<0.001
10%–49%	34 (27.0)	179 (29.1)	97 (41.8)	
<10%	80 (63.5)	403 (65.5)	101 (43.5)	
Neo‐adjuvant chemotherapy	17 (13.5)	96 (15.6)	54 (23.3)	0.015
Breast surgery				
Mastectomy	89 (70.6)	453 (73.7)	188 (81.0)	0.041
BCS	37 (29.4)	162 (26.3)	44 (19.0)	
Axillary surgery				
SLNB only	29 (23.0)	174 (28.3)	60 (25.9)	0.512
ALND	97 (77.0)	441 (71.7)	172 (74.1)	
Adjuvant therapy, *N* = 969				
Chemotherapy	64 (51.2)	321 (52.3)	200 (87.0)	<0.001
Endocrine therapy	103 (82.5)	536 (87.3)	105 (45.7)	<0.001
Radiation therapy	51 (40.8)	266 (43.3)	120 (52.2)	0.041

Abbreviations: ALND, axillary lymph node dissection; BCS, breast conversing surgery; CCI, Charlson Comorbidity Index; ER, estrogen receptor; HER2, human epidermal growth factor receptor 2; IDC, invasive ductal carcinoma; IHC, immunohistochemistry; LVI, lymph vascular invasion; PR, progestogen receptor; SLNB, sentinel lymph node biopsy; sTIL, stromal tumor‐infiltrating lymphocyte.

^a^ Thirty patients received a pathological complete response (pCR) after NAC.

Clinicopathologic features were compared according to HER2 status (Table 1). Age, histology, grade, tumor size, node status, ER, PR, and Ki67 statuses were differently distributed among HER2‐0, HER2‐low, and HER2‐positive tumors (all *p* < 0.05, Table 1). Multinomial logistic regression demonstrated that the overall distribution of node status (*p* = 0.014, Table 2), ER (*p* < 0.001), and PR (*p* = 0.001) had a significant difference according to HER2 status. Overall, HER2‐low tumors shared more similarity in clinicopathologic features with HER2‐0 ones. In addition, compared with HER2‐positive patients, HER2‐low ones were more likely to be ER‐positive (OR 3.64, 95% CI 2.02–5.78, *p* < 0.001) and PR‐positive (OR 2.08, 95% CI 1.33–3.48, *p* < 0.001), node‐negative (OR 1.49, 95% CI 1.11–2.94, *p* = 0.031), and with Ki67 <14% (OR 1.65, 95% CI 1.03–2.87, *p* = 0.047).

**TABLE 2:** Multivariate analysis of clinicopathological factors associated with HER2 status.

Characteristics	HER2‐Low			HER2+			*p* value
	OR	95% CI	*p* value^a^	OR	95% CI	*p* value^a^	
Age, year							
<55	0.72	0.46–1.12	0.142	1.02	0.61–1.69	0.831	0.066
≥55	1.00			1.00			
Histology							
IDC	0.45	0.06–3.92	0.492	3.21	0.63–6.16	0.255	0.121
Non‐IDC	1.00			1.00			
Grade							
I–II	1.60	1.00–2.63	0.050	1.24	0.71–2.16	0.648	0.057
III	1.00			1.00			
Tumor size, cm							
≤2	1.07	0.67–1.71	0.759	0.76	0.42–1.27	0.331	0.218
>2	1.00			1.00			
Lymph node status							
Negative	0.72	0.42–1.25	0.191	0.45	0.24–0.82	0.008	0.014
Positive	1.00			1.00			
ER status							
Positive	1.81	0.90–3.68	0.089	0.55	0.37–1.22	0.094	<0.001
Negative	1.00			1.00			
PR status							
Positive	1.04	0.59–1.82	0.640	0.37	0.25–0.74	0.032	0.001
Negative	1.00			1.00			
Ki67							
<14%	1.09	0.65–1.80	0.833	0.73	0.49–1.37	0.223	0.151
≥14%	1.00			1.00			
sTILs							
≥50%	0.60	0.29–1.25	0.237	0.96	0.42–2.19	0.914	0.020
10%–49%	1.34	0.78–2.31	0.148	1.24	0.52–2.91	0.157	
<10%	1.00			1.00			

Abbreviations: CI, confidence interval; ER, estrogen receptor; HER2, human epidermal growth factor receptor 2; IDC, invasive ductal carcinoma; LVI, lymph vascular invasion; OR, odds ratio; PR, progestogen receptor; sTIL, stromal tumor‐infiltrating lymphocyte.

^a^ Reference category: HER2‐0.

In ER‐positive subgroups, similarly, age (*p* = 0.011, Table S1), histology (*p* = 0.001), grade (*p* < 0.001), tumor size (*p* < 0.001), node status (*p* < 0.001), PR (*p* < 0.001), and Ki67 statuses (*p* < 0.001) were differently distributed among HER2‐0, HER2‐low, and HER2‐positive tumors. Besides, ER‐positive/HER2‐low patients had a significantly higher proportion of postmenopausal compared with ER‐positive/HER2‐0 and ER‐positive/HER2‐positive ones (*p* < 0.001). On the other hand, ER‐negative subgroups, only grade (*p* = 0.049) and PR status (*p* = 0.009), were differently distributed among patients with different HER2 statuses.

### 3.2 sTILs distribution and its impact factors by HER2 status

**FIGURE 1:**
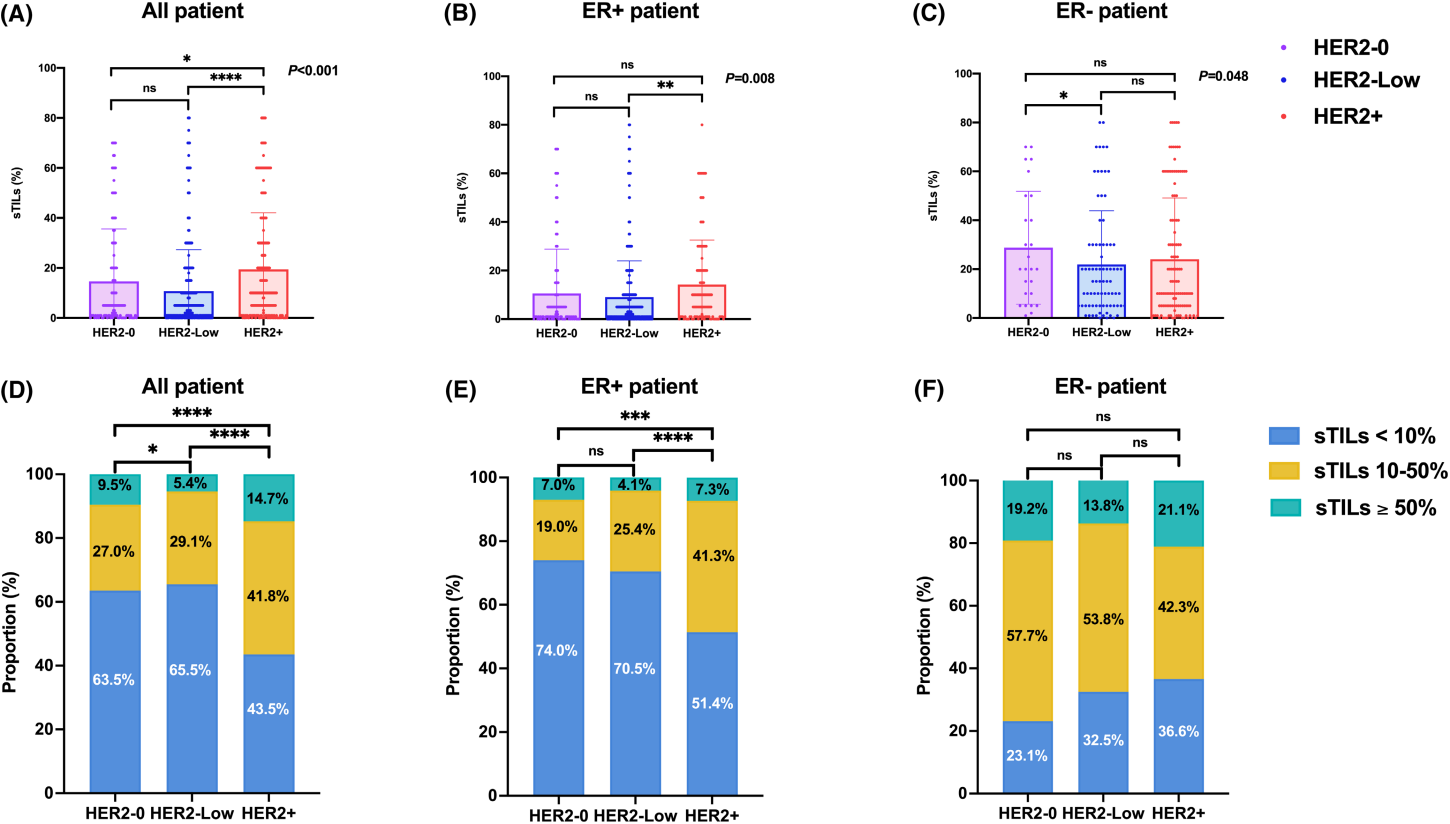
sTILs expression according to HER2 status. sTILs distribution in (A) all, (B) ER‐positive, and (C) ER‐negative population, and sTILs category distribution in (D) all, (E) ER‐positive, and (F) ER‐negative population. Boxplots and bars represent the mean value and standard deviation. *, *p* < 0.05. **, *p* <0.01. ***, *p* < 0.001. ****, *p* < 0.0001.

The percentage of sTILs was substantially different among HER2‐0, HER2‐low, and HER2‐positive tumors (univariate *p* < 0.001, Table 1; multivariate *p* = 0.020, Table 2), with mean proportions of 14.3%, 10.7%, and 19.4% in these three groups, respectively. HER2‐low tumors had a similar sTILs distribution to HER2‐0 cases (*p* = 0.064), both significantly lower than HER2‐positive ones (Figure 1A). Meanwhile, tumors with sTILs ≥50% accounted for the least proportion in HER2‐low cases (5.4%, vs. HER2‐0 9.5%, *p =* 0.048, vs. HER2‐positive 14.7%, *p* < 0.001, Figure 1D).

In addition, the difference in sTILs distribution existed regardless of ER status (Figure 1). For ER‐positive population, HER2‐0 and HER2‐low tumors shared similar levels of sTILs infiltration (*p* = 0.676), whereas HER2‐positive tumors had significantly increased sTILs (*p* < 0.001) compared with HER2‐low ones. For the ER‐negative population, sTILs level was significantly lower in HER2‐low tumors (mean value 22.0%) compared with HER2‐0 tumors (mean value 28.8%, *p* = 0.033), however, no significant difference in sTILs level was observed between HER2‐low and HER2‐positive cases (mean value 24.0%, *p* = 0.729).

**FIGURE 2:**
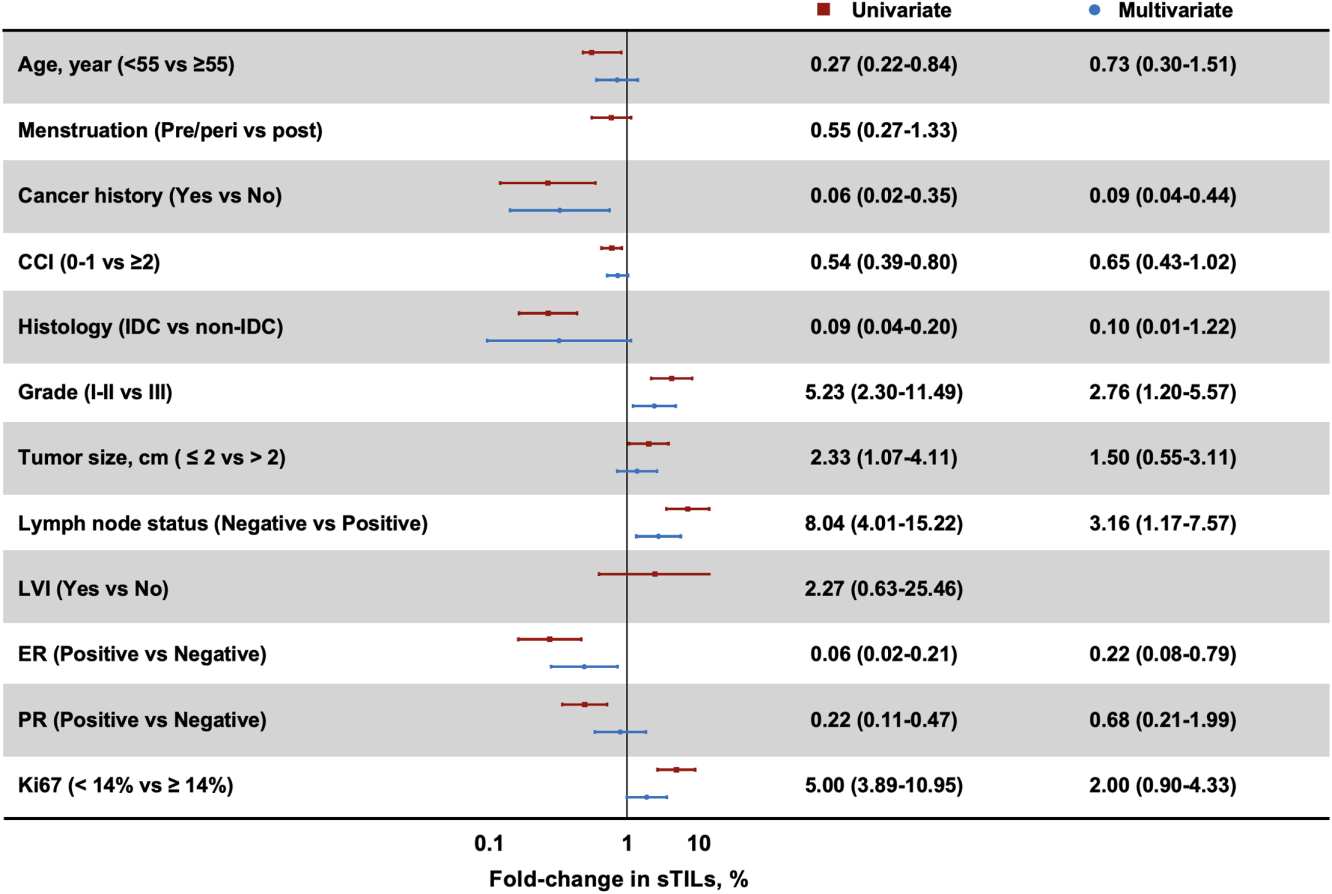
Univariable and multivariable analyses of factors associated with sTILs in HER2‐low population.

Next, we looked into the impact factors for sTILs infiltration. In the overall population, cancer history (OR 4.54, 95% CI 1.02–25.00, *p* = 0.007, Figure S2), high Charlson Comorbidity Index score (CCI, OR 1.45, 95% CI 1.02–2.08, *p* = 0.009), high histologic grade (OR 3.05, 95% CI 1.40–5.11, *p* = 0.004), lymph node involvement (OR 2.64, 95% CI 1.46–5.52, *p* < 0.001), and ER‐negative (OR 3.45, 95% CI 1.82–8.33, *p* < 0.001) were identified as significant predictors for high sTILs infiltration. Moreover, for HER2‐low patients, history of malignancy (OR 11.11, 95% CI 2.27–25.00, *p* = 0.003, Figure 2), grade III (OR 2.76, 95% CI 1.20–5.57, *p* = 0.008), node‐positive (OR 3.16, 95% CI 1.17–7.57, *p* < 0.001), and ER‐negative (OR 4.54, 95% CI 1.27–12.50, *p* < 0.001) tumors had a statistically higher level of sTILs infiltration.

### 3.3 Clinical outcomes of patients with different HER2 status

**FIGURE 3:**
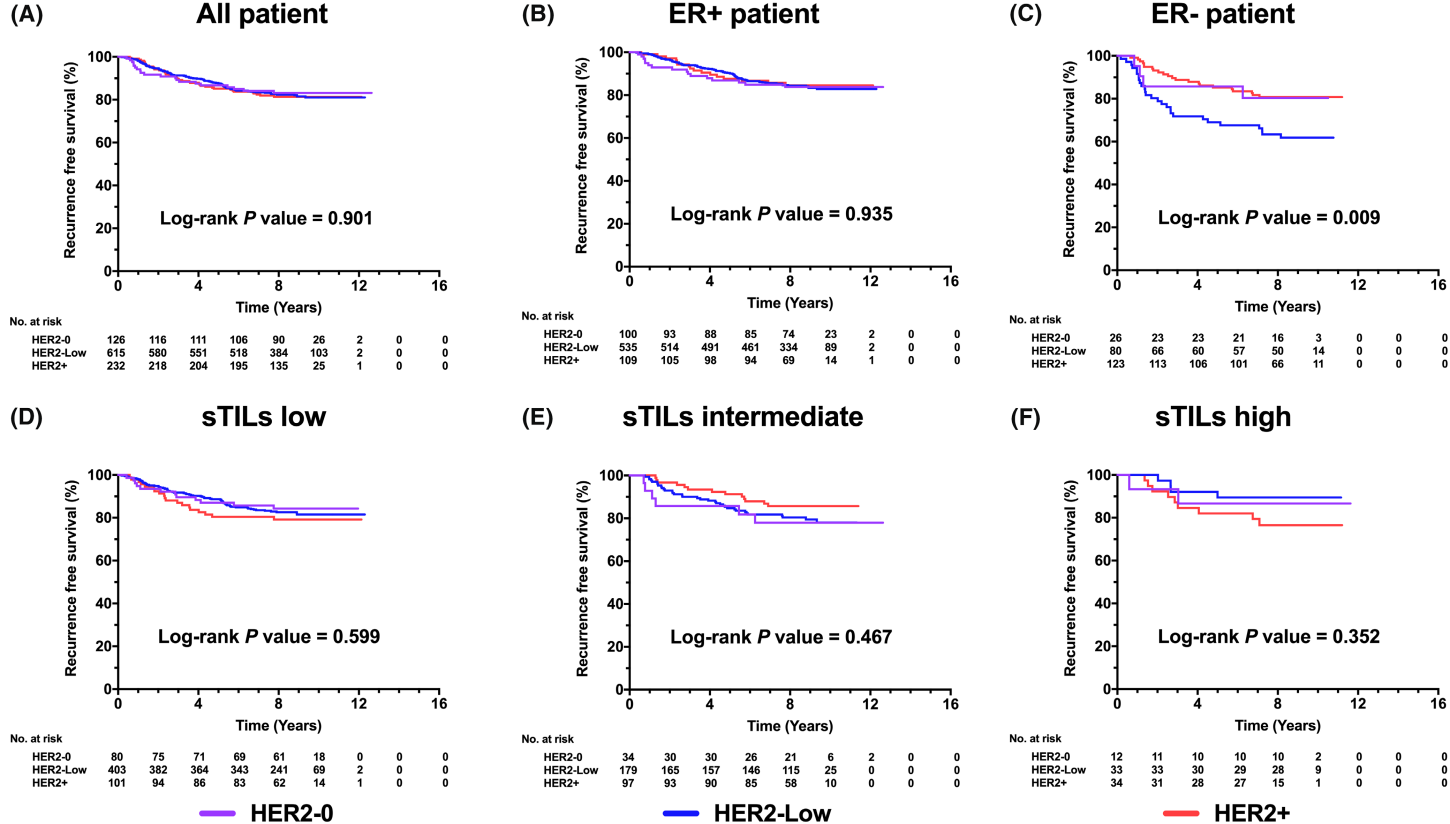
Prognostic value of HER2 status on recurrence‐free survival in (A) all, (B) ER‐positive, (C) ER‐negative, (D) sTILs low, (E) sTILs intermediate, and (F) sTILs high population.

After a median follow‐up of 104.47 (range 5.03–151.57) months, disease recurrence or death occurred in 173 cases. Different HER2 status was not associated with RFS (*p* = 0.901, Figure 3A, Table S2) or OS (*p* = 0.771, Figure S3A) in the overall population in the univariate model. The estimated 5‐year and 8‐year RFS were 86.7% and 83.2% for HER2‐0, 87.6% and 82.4% for HER2‐low, and 85.1% and 81.5% for HER2‐positive patients. After adjusted for clinicopathologic features including histologic grade, LVI, tumor size, node status, ER, PR, Ki67 level, and sTILs per 10% increment, no RFS difference was found between HER2‐low, HER2‐0, and HER2‐positive patients (*p* = 0.223; HER2‐low vs. HER2‐0, HR 0.97, 95% CI 0.57–1.64, *p* = 0.901; HER2‐low vs. HER2‐positive, HR 0.69, 95% CI 0.46–1.05, *p* = 0.085; HER2‐positive vs. HER2‐0, HR 1.12, 95% CI 0.39–3.25, *p* = 0.852, Table 3) in multivariate Cox regression model. Meanwhile, the estimated 5‐year and 8‐year OS was 89.2% and 87.5% for HER2‐0, 92.8% and 89.8% for HER2‐low, and 91.0% and 90.0% for HER2‐positive patients, respectively. After adjusted for clinicopathologic features, HER2 status was independently associated with OS (*p* = 0.014). HER2‐low patients had a comparable OS with HER2‐0 ones (HR 0.90, 95% CI 0.59–1.64, *p* = 0.534), but a significantly impaired OS compared with HER2‐positive ones (HR 2.17, 95% CI 1.23–3.85, *p* = 0.007).

**TABLE 3:** Multivariable Cox regression analysis of factors associated with prognosis in the whole population.

Characteristics	OS			RFS		
	HR	95% CI	*p* value	HR	95% CI	*p* value
Grade						
I–II	0.52	0.33–0.80	0.005	0.64	0.45–0.89	0.009
III	1.00			1.00		
LVI						
No	0.43	0.22–0.87	0.017	0.44	0.24–0.79	0.006
Yes	1.00			1.00		
Tumor size, cm						
≤2	0.53	0.34–0.83	0.005	0.53	0.38–0.75	<0.001
>2	1.00			1.00		
Lymph node status						
Negative	0.61	0.39–0.95	0.028	0.60	0.43–0.85	0.003
Positive	1.00			1.00		
ER						
Positive	0.32	0.20–0.54	<0.001	0.66	0.45–0.96	0.031
Negative	1.00			1.00		
PR						
Positive	0.63	0.36–1.11	0.111	0.82	0.53–1.27	0.378
Negative	1.00			1.00		
HER2 status			0.014			0.223
HER2‐0	1.11	0.61–1.70	0.534	0.97	0.57–1.64	0.901
HER2+	0.46	0.26–0.81	0.007	0.69	0.46–1.05	0.085
HER2‐Low	1.00			1.00		
Ki67						
<14%	0.90	0.53–1.52	0.684	1.09	0.74–1.61	0.663
≥14%	1.00			1.00		
sTILs per 10% increment	0.82	0.72–0.93	0.003	0.87	0.79–0.96	0.005

Abbreviations: CCI, Charlson Comorbidity Index; CI, confidence interval; ER, estrogen receptor; HER2, human epidermal growth factor receptor 2; HR, hazard ratio; LVI, lymph vascular invasion; OS, overall survival; PR, progestogen receptor; RFS, recurrence‐free survival; sTIL, stromal tumor‐infiltrating lymphocytes.

**FIGURE 4:**
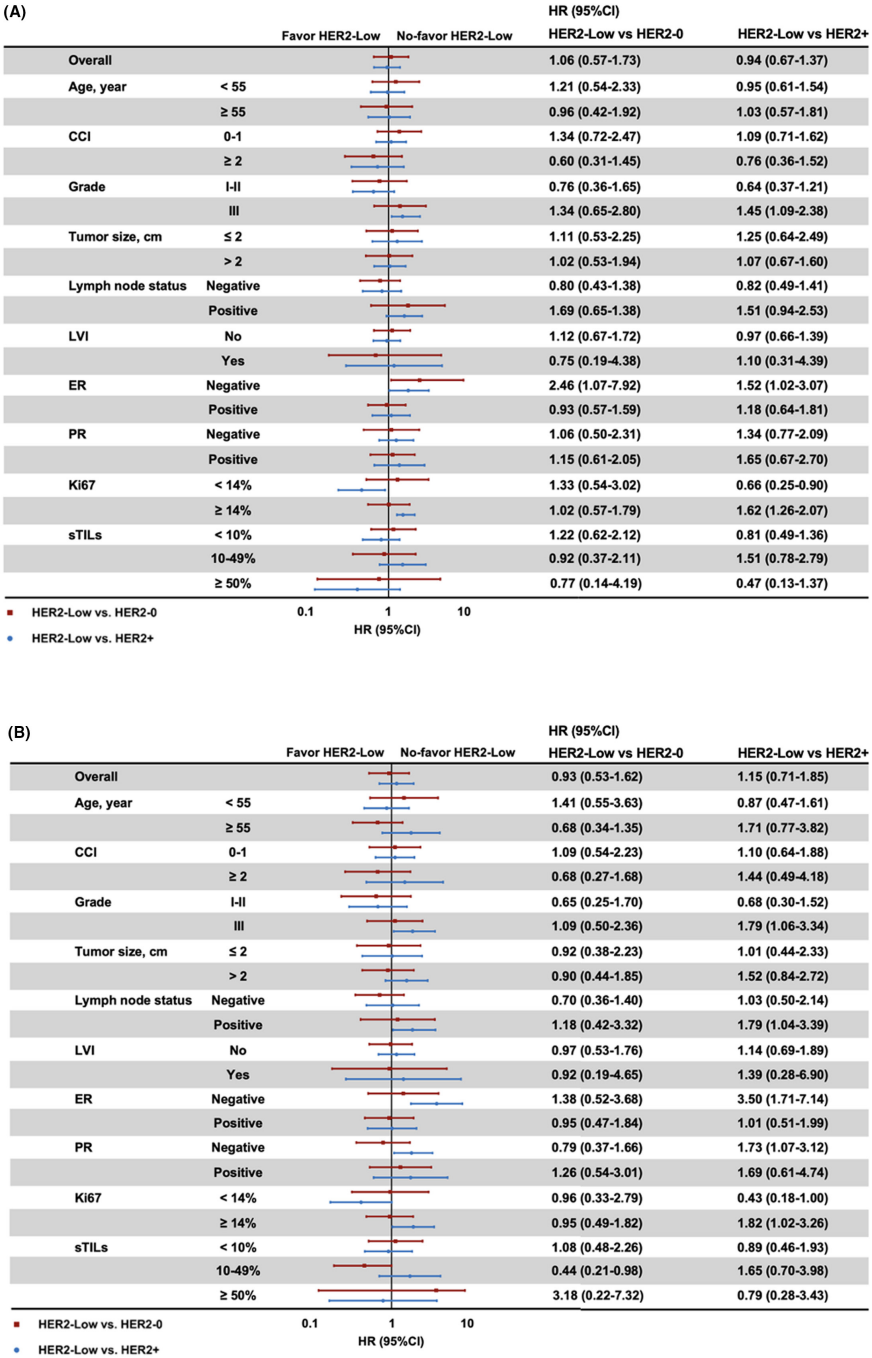
Subgroup prognostic analysis of HER2 status on (A) recurrence‐free survival and (B) overall survival.

ER status showed a tremendous impact on the prognosis of HER2‐low status (Figure 3, Figure S3, Table S8). For ER‐positive population, HER2‐low patients reported similar RFS to HER2‐0 and HER2‐positive ones (5‐year RFS, 86.9%, 90.1%, 87.6%; 8‐year RFS, 83.1%, 84.2%, 84.7%, *p* = 0.935, Figure 3). On the contrary, for ER‐negative population, HER2‐low patients showed significantly impaired RFS (5‐year 69.0%, 8‐year 66.1%) compared with HER2‐0 (5‐year 85.7%, 8‐year 80.4%, hazard ratio [HR] 2.46, 95% CI 1.07–7.92, *p* < 0.001, Figure 4A) or HER2‐positive ones (5‐year 82.9%, 8‐year 78.5%, HR 1.52, 95% CI 1.02–3.07, *p* < 0.001). No significant OS difference was observed among ER‐positive/HER2‐0, ER‐positive/HER2‐low, and ER‐positive/HER2‐positive patients (5‐year OS, 91.9%, 95.6%, 93.3%; 8‐year OS, 90.1%, 92.2%, 90.9%, *p* = 0.997, Figure 4B). Nevertheless, in ER‐negative patients, the estimated 5‐year and 8‐year OS was 71.8% and 70.4% for HER2‐low cohort, similar to HER2‐0 (5‐year 76.2%, 8‐year 71.4%, HR 1.38, 95% CI 0.52–3.68, *p* = 0.234), but significantly worse than HER2‐positive ones (5‐year 88.9%, 8‐year 88.9%, HR 3.50, 95% CI 1.71–7.14, *p* < 0.001).

For HER2‐low patients, histologic grade (univariate, grade I–II, 5‐year 92.7%, 8‐year 87.7%, vs. grade III, 5‐year 78.1%, 8‐year 73.4%, *p* < 0.001, Table S3; multivariate, HR 0.52, 95% CI 0.34–0.80, *p* = 0.003, Table S4), tumor size (univariate, ≤2 cm, 5‐year, 91.3%, 8‐year 87.7%, vs. >2 cm, 5‐year 81.6%, 8‐year 73.9%, *p* < 0.001; multivariate, HR 0.62, 95% CI 0.41–0.96, *p* = 0.030), nodal status (univariate, negative, 5‐year 90.8%, 8‐year 87.5%, vs. positive, 5‐year 78.3%, 8‐year 67.7%, *p* < 0.001; multivariate, HR 0.46, 95% CI 0.30–0.70, *p* < 0.001), ER negativity (univariate, 5‐year 69.0%, 8‐year 67.6%, vs. positive, 5‐year 90.1%, 8‐year 84.4%, *p* < 0.001; multivariate, HR 2.86, 95% CI 1.61–5.08, *p* = 0.003) and sTILs per 10% increment (HR 0.82, 95% CI 0.70–0.95, *p* = 0.008) were independently associated with RFS. Similarly, sTILs per 10% increment (HR 0.79, 95% CI 0.62–0.90, *p* = 0.007) were also independently associated with OS in the multivariate Cox regression model.

### 3.4 Clinical outcomes of HER2‐low patients according to different sTILs level

To note, as aforementioned data, per 10% increment of sTILs was related to a 19% risk reduction of disease relapse and 21% risk of death in the HER2‐low population, making it one of the most important independent prognostic factors in the HER2‐low population. Subgroup exploration found that in ER‐negative/HER2‐low population, per 10% increment in sTILs brought 33% decrease in OS risk (univariate, *p* = 0.004, Table S5; multivariate, HR 0.67, 95% CI 0.50–0.92, *p* = 0.006, Table S6) and 31% decrease in RFS risk (univariate, *p* = 0.001; multivariate, HR 0.69, 95% CI 0.52–0.94, *p* = 0.010). On the other hand, sTILs increment showed no significant association with OS (*p* = 0.289) or RFS (*p* = 0.342) in ER‐positive/HER2‐low ones.

To further explore the prognostic value of sTILs in the specific population, we then classified sTILs into low infiltrate (<10%), intermediate infiltrate (10%–49%), and high infiltrate (≥50%) categories (Table S9). No significant relationship between sTILs infiltration level and RFS (low infiltrate, 5‐year 88.7%, 8‐year 82.6%; intermediate infiltrate, 5‐year 84.7%, 8‐year 80.4%; high infiltrate, 5‐year 89.5%, 8‐year 89.5%, *p* = 0.344, Figure S4) or OS (low infiltrate, 5‐year 93.1%, 8‐year 89.7%; intermediate infiltrate, 5‐year 92.4%, 8‐year 89.4%; high infiltrate, 5‐year 92.1%, 8‐year 92.1%, *p* = 0.663) was observed in HER2‐low patients. Furthermore, we found that the prognostic value of the sTILs category in this population is also dependent on ER status. In ER‐positive/HER2‐low population, we constantly found no survival difference among different sTILs infiltration groups (RFS, low infiltrate, 5‐year 90.8%, 8‐year 84.6%; intermediate infiltrate, 5‐year 87.9%, 8‐year 82.3%; high infiltrate, 5‐year 92.0%, 8‐year 92.0%, *p* = 0.318; OS, low infiltrate, 5‐year 95.1%, 8‐year 91.8%; intermediate infiltrate, 5‐year 97.0%, 8‐year 93.2%; high infiltrate, 5‐year 96.0%, 8‐year 96.0%, *p* = 0.669), while, in ER‐negative/HER2‐low population, higher sTILs level was proved significantly associated with superior RFS (low infiltrate, 5‐year 50.0%, 8‐year 45.0%; intermediate infiltrate, 5‐year 73.7%, 8‐year 73.7%; high infiltrate, 5‐year 84.6%, 8‐year 84.6%, *p* = 0.014) and OS (low infiltrate, 5‐year 55.0%, 8‐year 43.8%; intermediate infiltrate, 5‐year 76.3%, 8‐year 76.3%; high infiltrate, 5‐year 84.6%, 8‐year 84.6%, *p* = 0.020).

Additionally, the influence of HER2 status on prognosis in different sTILs infiltration categories was also examined (Figure 3, Figure S4). No difference in survival was reported between HER2‐0, HER2‐low, and HER2‐positive groups of patients with low or high sTILs infiltration (all *p* > 0.05). However, in intermediate sTILs infiltration group, HER2‐0 patients had the significantly worst OS compared with HER2‐low and HER2‐positive patients (HER2‐0, 5‐year 78.6%, 8‐year 75.0%; HER2‐low, 5‐year 92.4%, 8‐year 89.4%; HER2‐positive, 5‐year 94.5%, 8‐year 91.9%, *p* = 0.042, Figure S3).

## 4 DISCUSSION

To our knowledge, this is the first report focusing on the role of immune cell infiltration in HER2‐low breast cancer. Our study not only provides a comprehensive overview of the clinicopathologic features of HER2‐low disease but also establishes the distribution feature and prognostic value of sTILs in this newly raised population. According to our results, HER2‐low patients shared more similarities with HER2‐0 cases in clinicopathologic features, including sTILs infiltration, significantly different from HER2‐positive ones. sTILs infiltration is an independent favorable prognostic factor in HER2‐low patients, whose prognostic value was more notable in ER‐negative/HER2‐low population.

As anticipated, the differences in tumor biology distribution to HER2 protein expression and ER status are consistent with previous reports.^9^, ^34^ Patients with HER2‐low status were more often with low clinical risk, for example, more low‐grade, ER‐positive, PR‐positive, and low Ki67 tumors, compared with HER2‐positive ones. However, we found HER2‐low a population very much similar to HER2‐0 patients, which was different from the declaration by Alexander et al.^35^ HER2‐low cancer seems to have a little higher degree of *ERBB2* gene amplification.^9^ In a recently published article, Agostinetto et al.^8^ observed a higher proportion of HER2‐enriched intrinsic subtype tumors in the HER2‐low cohort compared with HER2‐0 ones, but further exploration suggested that *ERBB2* is not the principal oncological driver in this population.^9^ This TCGA data set‐based research might give us a transcriptomic‐level explanation of the similarity between the HER2‐low and HER2‐0 cohort.

Despite not many differences in tumor biology between HER2‐low and HER2‐0 populations, HER2 protein expression still showed a plausible effect on prognosis,^36^ especially in ER‐negative patients. Hitherto, limited data had been reported about the influence of HER2‐low status on long‐term survival, while, unfortunately, with conflicting results. Jacot et al. declared in the analysis of 296 clinically TN breast cancer patients that patients with HER2 2+ tumor had a worse prognosis than HER2 0 or 1+ ones.^37^ In contrast to our discovery, in a pooled analysis of four prospective clinical trials including 2,310 early breast cancer patients, Denkert et al. demonstrated that HER2‐low patients had significantly longer survival than HER2‐0 patients.^10^ Meanwhile, in other larg‐scale retrospective analyses, investigators found no difference in survival between HER2‐low and HER2‐0 patients, irrespective of ER status.^38^, ^39^, ^40^ Selection bias may be a reason for the controversy. What is more, discrepancy in interpretation standard of HER2 status and definition of HER2‐low status might explain part of the differences. The initial ASCO/CAP guideline for HER2 test was published in 2007^41^ and was then updated in 2013^42^ and 2018,^5^ while there is still no gold standard for HER2‐low tumor nowadays. Some of the previous studies adopted different definitions of HER2‐low status in one analysis which will no doubt lead to the unstable conclusion. Fernandez et al. demonstrated in a recent brief report the poor concordance across different laboratories and pathologists for ERBB2 IHC scoring (26% between 0 and 1+ and 58% between 2+ and 3+) by using a current standard ERBB2 IHC assay,^43^ and as we described, we adopted the same 2018 ASCO/CAP standard to define HER2 status and each result was accomplished by at least two independent pathologists in order to avoid the possible error, nevertheless, the potential bias in HER2 scoring by IHC array might be inevitable. Besides, further exploration of the better criterion of HER2‐low status by adding genomic and transcriptome information is on the way.

Crosstalk between ER and HER2 signaling pathways has been previously established.^44^ And clinically, we observed a marvelous impression of ER status on biological characteristics and prognosis of HER2‐low tumor. More ER‐positive patients were discovered in HER2‐low group, consistent with previous articles.^9^, ^45^ Besides, HER2‐low was proven related to unfavorable prognosis only in the ER‐negative subgroup, which agreed with Guven's conclusion^46^ not different phenomenon was also noticed in several other studies.^38^, ^39^ The interaction effect of ER status on the prognosis value of HER2 status was still lack of consensus.

In our study, we specifically evaluated the role of sTILs in HER2‐low populations. sTILs have been well studied in the last decade with the development of antitumor immunotherapy. Many published articles revealed that lymphocytes infiltrate differently in different subtypes of breast cancer,^47^ in detail, a higher proportion of sTILs were easily observed in ER‐negative tumors,^18^, ^48^ but ER‐positive tumors showed heterogeneous and low‐density lymphocyte infiltration in the stromal area.^18^ Here, we first reported the sTILs distribution in HER2‐low breast cancer and we found that sTILs in HER2‐low tumors are significantly lower than HER2‐positive ones and numerically lower than HER2‐0 ones. When taking ER status into account, the same circumstance was noticed in ER‐positive patients, nevertheless, in ER‐negative patients, HER2‐low had a significantly lower sTILs level than HER2‐0 patients and numerically lower versus HER2‐positive ones. HER2‐low population maintained the lowest infiltration level of sTILs among different HER2 statuses regardless of ER status, which might explain why HER2‐low patients have the worst prognosis. Our finding is in line with recently published research that ER‐negative/HER2‐low tumors contained significantly lower sTILs level than HER2‐0 ones.^40^ And this may also be an explanation for why traditional anti‐HER2 monoclonal antibodies demonstrate poor efficacy in HER2‐low tumors since the low immune cells infiltration restrict the ADCC and ADCP which are the major antitumor mechanism of trastuzumab.^13^

sTILs were described as a prognostic factor of breast cancer in many studies.^49^ Previous research revealed that the prognostic value of sTILs varies according to different molecular subtypes.^50^ A meta‐analysis involving 33 studies showed that higher sTILs were associated with improved survival in HER2‐positive and TN breast cancer patients,^51^ while other researchers declared sTILs as a negative prognostic factor in HoR‐positive breast cancer.^20^, ^47^ HER2‐low was a newly raised population, as a potential target user of novel ADCs and immunotherapy. Novel ADCs, such as DS–8201 and SYD985, showed encouraging response rates in HER2‐low metastatic breast cancer patients in Phase 1 trials,^52^, ^53^ and the efficacy and safety of DS–8201 in unresectable and/or metastatic HER2‐low breast cancer patients have been proven in DESTINY‐Breast04.^14^ In a Phase 1b study, the combination of DS–8201 and the anti‐PD‐1 agent nivolumab demonstrated considerable efficacy and manageable safety profile in HER2‐low patients, therefore, several trials adapting the combination strategy of anti‐HER2 therapy and immunotherapy in the HER2‐low population are ongoing (ClinicalTrials.gov Identifier: NCT04042701, NCT03524572, NCT03742102, NCT04556773). But we still knew little about the prognosis value of sTILs in HER2‐low population. Herein, we investigated the influence of sTILs on survival in HER2‐low patients and we noticed that the prognostic value of sTILs differs according to ER status. To note, higher sTILs infiltration inferred better prognosis only in ER‐negative/HER2‐low patients but not ER‐positive/HER2‐low ones. Plentiful analyses clarified that ER status is a vital impact factor on the prognostic value of sTILs.^20^, ^51^, ^52^ According to our data, in ER‐positive patients, the HER2‐low population seemed to be more similar to HER2‐0 ones. On the other hand, in ER‐negative patients, we found HER2‐low the only subgroup in whom sTILs could influence survival. Triple‐negative breast cancer (TNBC), in whom it has been proven that sTILs correlate with response to novel immunotherapy and disease outcomes.^54^ As a subset of TNBC, our data confirmed the conclusion in ER‐negative/HER2‐low tumors. However, in the ER‐negative/HER2‐0 subgroup, we only observed a trend of favorable survival in high sTILs patients, probably due to a relatively small number of patients (N = 120). Although previous studies declared that sTILs were related to superior survival in HER2‐positive breast cancer,^16^, ^17^, ^19^, ^23^, ^24^ we found no such relationship in our study. Relatively short follow‐up,^19^ disparate study endpoint, for instance, ShortHER trial used distant disease‐free survival,^24^ and different treatments especially different in the use of trastuzumab^16^, ^23^, ^24^ might explain the controversial results in other previous studies.

The main limitation of the current study includes the small sample size, particularly in subgroup analysis, the retrospective character, and the imbalance of patients of different HER2 statuses. sTILs assessment data of a certain number of patients (*N* = 2,014, Table S7) were not available due to the retrospective nature. In particular, there is no significant difference in ER status, HER2 status, and surgical and systemic treatment features between patients with and without sTILs data, while, more premenopausal patients and patients with high Ki67 levels had no sTILs data which may lead to potential bias. Further prospective research in a larger population is therefore necessary. Besides, as the patients in our study were treated from 2009 to 2013, these patients may have received outdated therapies and may not get benefit from more recent advancements such as multigene assays, de‐escalation of axillary lymph node dissection, and novel antibody‐drug conjugates, which may cause considerable bias in disease outcomes of these patients. Additionally, the number of patients undergoing NAC was too small for further subgroup analysis on the impact of NAC on the sTILs infiltration for patients with different HER2 statuses. Although our post‐hoc analysis showed similar sTILs distribution in NAC patients (Table S10), with no statistical variation in sTILs after NAC (Figure S5), further investigation of immune microenvironment in this group of patients is on the way. What is more, there is still no standard definition in HER2‐low status, and no consensus on the cutoff value of sTILs has been reached, either. To note, we did not address intratumor heterogeneity of HER2 expression, which potentially occurs in 1%–34% of all breast cancer patients and may interfere with prognosis.^55^ Finally, all our research is only based on the IHC test and protein level, further translational research based on novel gene expression assays and multi‐omics analysis is warranted to provide us a more detailed view of HER2‐low tumor.

In our current study, we found that HER2‐low breast cancer patients had similar clinicopathological parameters with HER2‐0 rather than HER2‐positive cases, had relatively low sTILs, and had significantly inferior survival in the ER‐negative subgroup. In the ER‐negative/HER2‐low population, higher sTILs infiltration was independently associated with favorable survival. Our findings suggested a potential clinical benefit by increasing sTILs infiltration and the activation of tumor immune microenvironment by using novel therapeutic agents such as ADCs and immunotherapy, especially in those ER‐negative/HER2‐low, who had significantly inferior survival, warranting further exploration and translational analysis in clinical use.

## AUTHOR CONTRIBUTIONS

**Yujie Lu:** Conceptualization (equal); data curation (equal); formal analysis (equal); investigation (equal); methodology (equal); software (equal); visualization (equal); writing – original draft (lead). **Yiwei Tong:** Funding acquisition (equal); methodology (equal); project administration (equal); software (equal); writing – review and editing (lead). **Xiaochun Fei:** Data curation (equal); methodology (equal); resources (equal); validation (equal); writing – review and editing (supporting). **Xiaosong Chen:** Conceptualization (equal); funding acquisition (equal); project administration (equal); resources (lead); supervision (lead); validation (equal); writing – review and editing (supporting). **Kunwei Shen:** Conceptualization (equal); funding acquisition (equal); resources (equal); supervision (equal); validation (equal); writing – review and editing (supporting).

## FUNDING INFORMATION

The authors received financial support from the National Natural Science Foundation of China (grant number: 81772797, 82072897) and Shanghai Sailing Program (grant number: 21YF1427400). All these financial sponsors had no role in the study design, data collection, analysis, or interpretation.

## CONFLICT OF INTEREST STATEMENT

The authors declare no competing interests.

## ETHICS STATEMENT

This study was approved by the independent Ethical Committees of Ruijin Hospital, Shanghai Jiao Tong University School of Medicine. All procedures were in accordance with the ethical standards of the institutional and/or national research committee and with the 1964 Helsinki declaration and its later amendments or comparable ethical standards.

## Supporting information


Figure S1.
Figure S2.
Figure S3.
Figure S4.
Figure S5.

Table S1.
Table S2.
Table S3.
Table S4.
Table S5.
Table S6.
Table S7.
Table S8.
Table S9.
Table S10.

## Data Availability

The data analyzed in the current study are available from the corresponding authors upon reasonable request.
