# Supplementary material for: Clinical characteristics, tumor‐infiltrating lymphocytes, and prognosis in HER2‐low breast cancer: A comparison study with HER2‐zero and HER2‐positive disease
Source: Cancer Med. 2023 Jun 27;12(15):16264–78. doi: 10.1002/cam4.6290 (PMC10469737; doi:10.1002/cam4.6290)
Supplement: Supplementary file 1 — Figure S1. Figure S2. Figure S3. Figure S4. Figure S5. [file CAM4-12-16264-s002.pptx]

## Slide 1
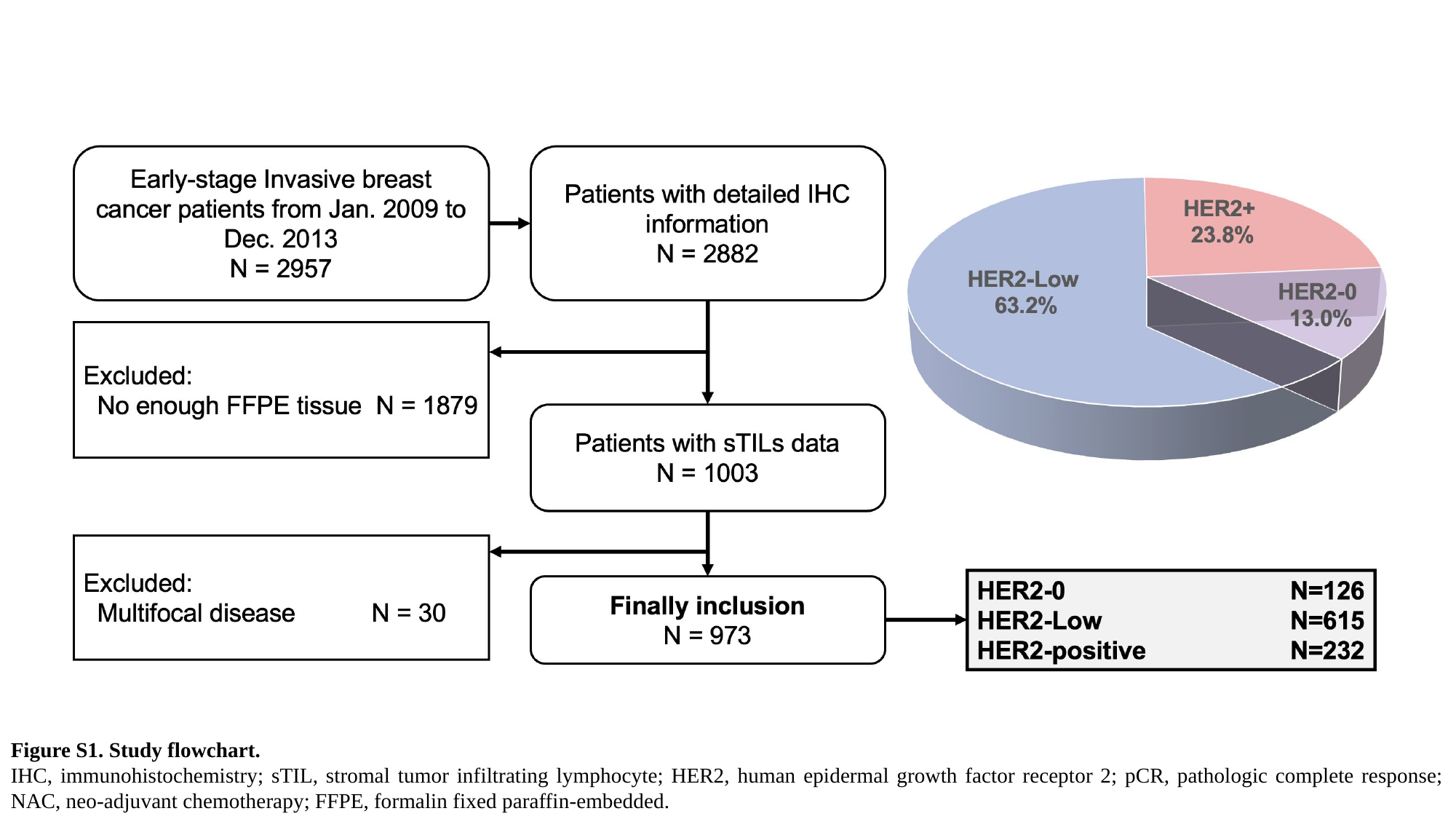

Figure S1. Study flowchart.
IHC, immunohistochemistry; sTIL, stromal tumor infiltrating lymphocyte; HER2, human epidermal growth factor receptor 2; pCR, pathologic complete response; NAC, neo-adjuvant chemotherapy; FFPE, formalin fixed paraffin-embedded.

## Slide 2
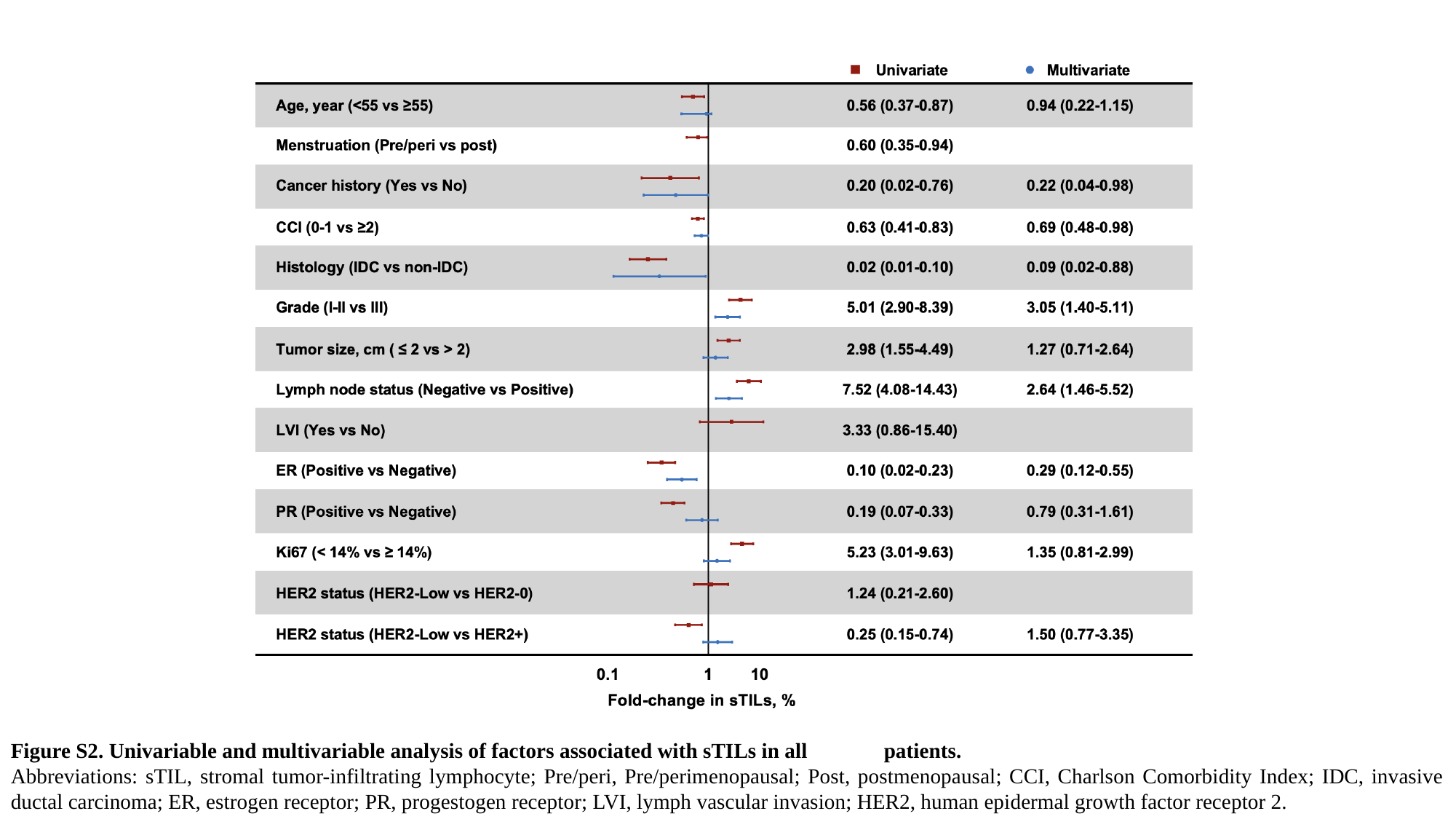

Figure S2. Univariable and multivariable analysis of factors associated with sTILs in all 	patients.
Abbreviations: sTIL, stromal tumor-infiltrating lymphocyte; Pre/peri, Pre/perimenopausal; Post, postmenopausal; CCI, Charlson Comorbidity Index; IDC, invasive ductal carcinoma; ER, estrogen receptor; PR, progestogen receptor; LVI, lymph vascular invasion; HER2, human epidermal growth factor receptor 2.

## Slide 3
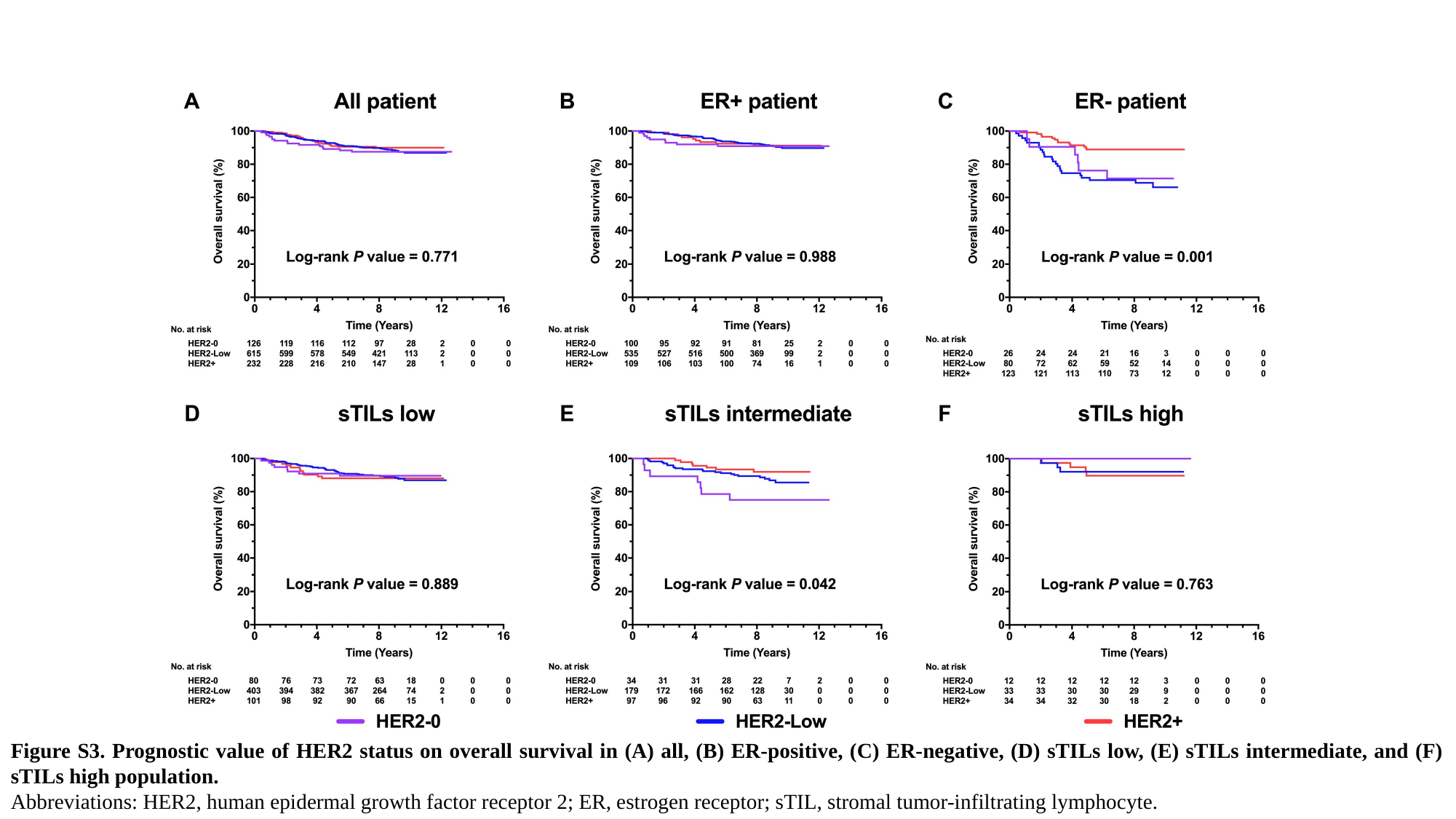

Figure S3. Prognostic value of HER2 status on overall survival in (A) all, (B) ER-positive, (C) ER-negative, (D) sTILs low, (E) sTILs intermediate, and (F) sTILs high population.
Abbreviations: HER2, human epidermal growth factor receptor 2; ER, estrogen receptor; sTIL, stromal tumor-infiltrating lymphocyte.

## Slide 4
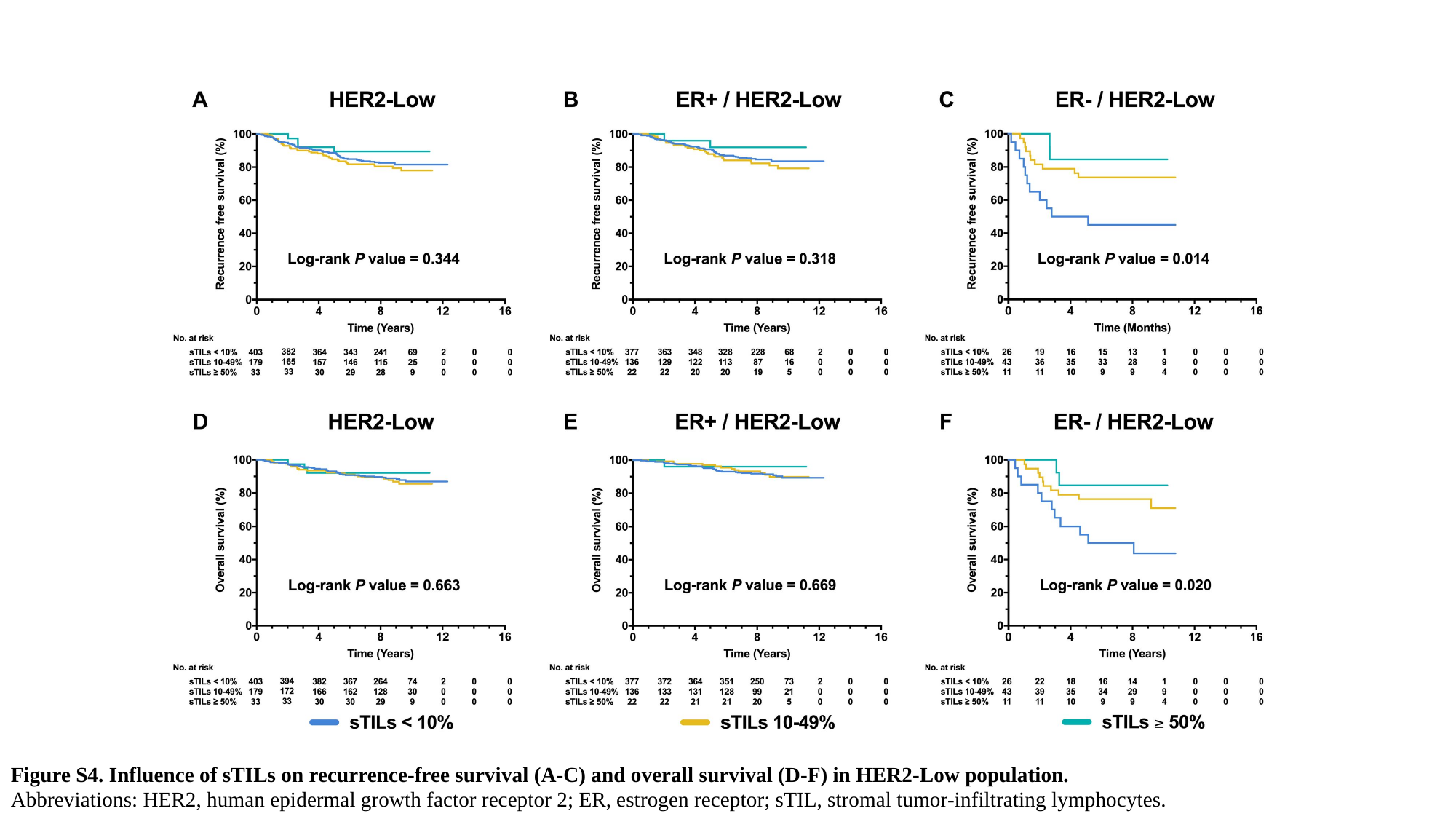

Figure S4. Influence of sTILs on recurrence-free survival (A-C) and overall survival (D-F) in HER2-Low population.
Abbreviations: HER2, human epidermal growth factor receptor 2; ER, estrogen receptor; sTIL, stromal tumor-infiltrating lymphocytes.

## Slide 5
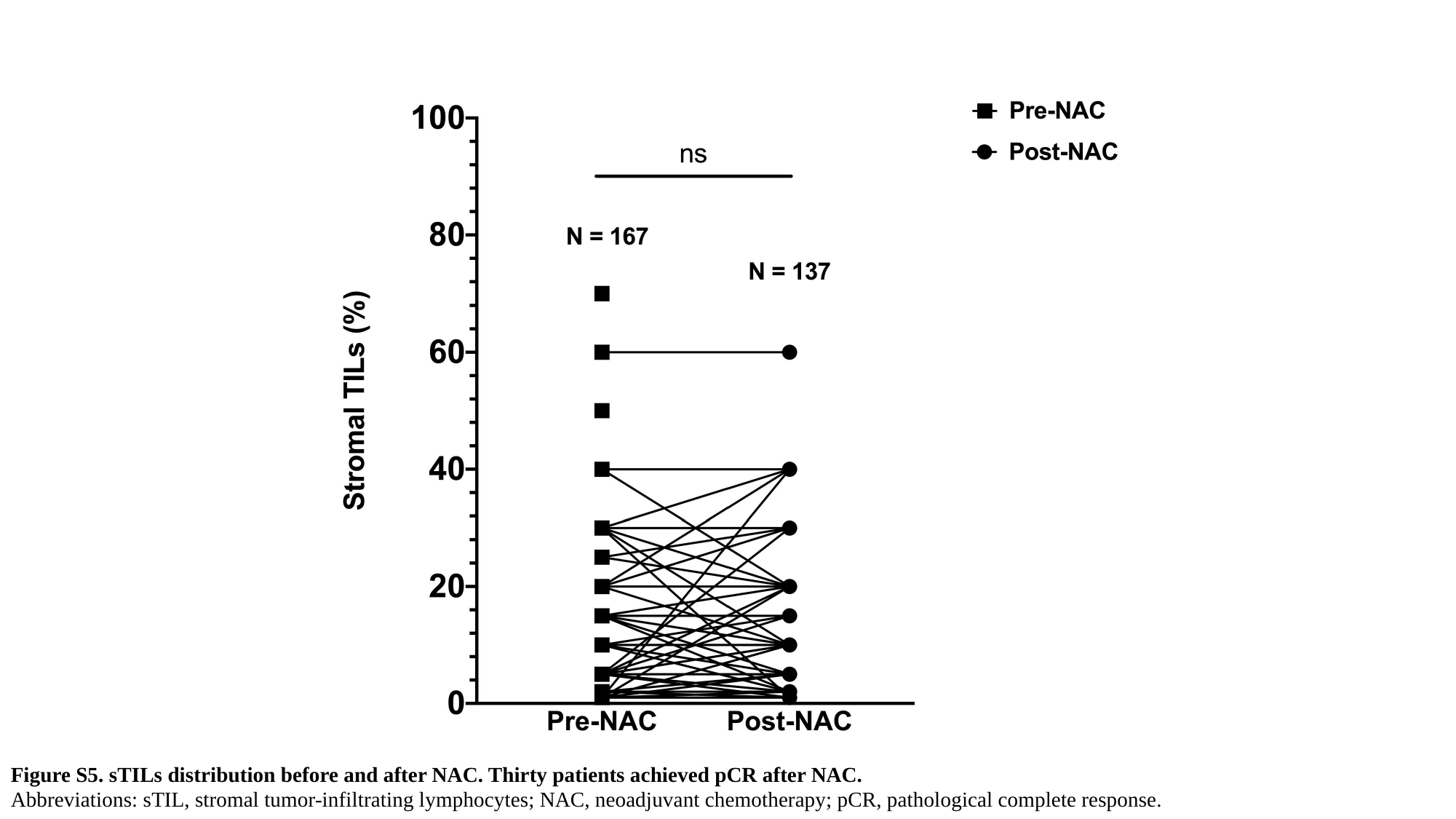

Figure S5. sTILs distribution before and after NAC. Thirty patients achieved pCR after NAC.
Abbreviations: sTIL, stromal tumor-infiltrating lymphocytes; NAC, neoadjuvant chemotherapy; pCR, pathological complete response.
