# Supplementary material for: Clinical characteristics, tumor‐infiltrating lymphocytes, and prognosis in HER2‐low breast cancer: A comparison study with HER2‐zero and HER2‐positive disease
Source: Cancer Med. 2023 Jun 27;12(15):16264–78. doi: 10.1002/cam4.6290 (PMC10469737; doi:10.1002/cam4.6290)
Supplement: Supplementary file 2 — Table S1. Table S2. Table S3. Table S4. Table S5. Table S6. Table S7. Table S8. Table S9. Table S10. [file CAM4-12-16264-s001.docx]

**Table S1. Baseline characteristics stratified ER and HER2 status.**

| Characteristics | ER-positive | | | | ER-negative | | | |
| --- | --- | --- | --- | --- | --- | --- | --- | --- |
|  | HER2-0  N=100 (%) | HER2-Low N=535 (%) | HER2+  N=109 (%) | *P* value | HER2-0  N=26 (%) | HER2-Low N=80 (%) | HER2+  N=123 (%) | *P* value |
| Age, years (median, range) | 53.0 (27-86) | 57.0 (24-92) | 50.0 (23-90) | <0.001 | 54.0 (42-77) | 53.0 (28-83) | 54.0 (29-81) | 0.503 |
| Age, years |  |  |  | 0.011 |  |  |  | 0.826 |
| < 55 | 54 (54.0) | 238 (44.5) | 64 (58.7) |  | 15 (57.7) | 47 (58.8) | 67 (54.5) |  |
| ≥ 55 | 46 (46.0) | 297 (55.5) | 45 (41.3) |  | 11 (42.3) | 33 (41.3) | 56 (45.5) |  |
| Menstruation |  |  |  | <0.001 |  |  |  | 0.158 |
| Pre/perimenopausal | 45 (45.0) | 197 (36.8) | 63 (57.8) |  | 10 (38.5) | 36 (45.0) | 39 (31.7) |  |
| Postmenopausal | 55 (55.0) | 338 (63.2) | 46 (42.2) |  | 16 (61.5) | 44 (55.0) | 84 (68.3) |  |
| Prior cancer history |  |  |  | 0.690 |  |  |  | 0.635 |
| Yes | 5 (5.0) | 23 (4.3) | 3 (2.8) |  | 0 (0.0) | 2 (2.5) | 4 (3.3) |  |
| No | 95 (95.0) | 512 (95.7) | 106 (97.2) |  | 26 (100.0) | 78 (97.5) | 118 (96.7) |  |
| CCI |  |  |  | 0.023 |  |  |  | 0.764 |
| 0-1 | 67 (67.0) | 356 (66.5) | 87 (79.8) |  | 20 (76.9) | 62 (77.5) | 90 (73.2) |  |
| ≥ 2 | 33 (33.0) | 179 (33.5) | 22 (20.2) |  | 6 (23.1) | 18 (22.5) | 33 (26.8) |  |
| Histology* |  |  |  | 0.001 |  |  |  | 0.092 |
| IDC | 81 (81.8) | 464 (87.7) | 104 (98.1) |  | 21 (100.0) | 65 (91.5) | 114 (97.4) |  |
| non-IDC | 18 (18.2) | 65 (12.3) | 2 (1.9) |  | 0 (0.0) | 6 (8.5) | 3 (2.6) |  |
| Grade* |  |  |  | <0.001 |  |  |  | 0.049 |
| I-II | 48 (48.5) | 332 (62.8) | 51 (48.1) |  | 2 (9.5) | 23 (32.4) | 39 (33.3) |  |
| III | 26 (26.3) | 135 (25.5) | 49 (46.2) |  | 19 (90.5) | 37 (52.1) | 78 (66.7) |  |
| Unknown | 25 (25.2) | 62 (11.7) | 6 (5.7) |  | 0 (0.0) | 11 (15.5) | 0 (0.0) |  |
| Tumor size, cm* |  |  |  | <0.001 |  |  |  | 0.262 |
| ≤ 2 | 69 (69.7) | 337 (63.7) | 45 (42.5) |  | 6 (28.6) | 32 (45.1) | 56 (47.9) |  |
| > 2 | 30 (30.3) | 192 (36.3) | 61 (57.5) |  | 15 (71.4) | 39 (54.9) | 61 (52.1) |  |
| Lymph node status* |  |  |  | <0.001 |  |  |  | 0.458 |
| Negative | 90 (90.9) | 412 (77.9) | 55 (51.9) |  | 9 (42.9) | 36 (50.7) | 66 (56.4) |  |
| Positive | 9 (9.1) | 117 (22.1) | 51 (48.1) |  | 12 (57.1) | 35 (49.3) | 51 (43.6) |  |
| LVI* |  |  |  | 0.395 |  |  |  | 0.165 |
| Yes | 4 (4.0) | 10 (1.9) | 2 (1.9) |  | 1 (4.8) | 8 (11.3) | 5 (4.3) |  |
| No | 95 (96.0) | 519 (98.1) | 104 (98.1) |  | 20 (95.2) | 63 (88.7) | 112 (95.7) |  |
| PR status |  |  |  | <0.001 |  |  |  | 0.009 |
| Positive | 85 (85.0) | 449 (83.9) | 73 (67.0) |  | 0 (0.0) | 12 (15.0) | 6 (4.9) |  |
| Negative | 15 (15.0) | 86 (16.1) | 36 (33.0) |  | 26 (100.0) | 68 (85.0) | 117 (95.1) |  |
| Ki67 |  |  |  | <0.001 |  |  |  | 0.086 |
| < 14% | 52 (52.0) | 274 (51.2) | 32 (29.4) |  | 2 (7.7) | 17 (21.2) | 14 (11.4) |  |
| ≥ 14% | 48 (48.0) | 261 (48.8) | 77 (70.6) |  | 24 (92.3) | 63 (78.8) | 109 (88.6) |  |
| sTILs (mean, range) | 10.5% (0-70) | 9.0% (0-80) | 14.2% (0-80) | 0.008 | 28.8% (2-70) | 22.0% (0-80) | 24.0% (0-80) | 0.048 |
| sTILs categories |  |  |  | 0.001 |  |  |  | 0.335 |
| ≥ 50% | 7 (7.0) | 22 (4.1) | 8 (7.3) |  | 5 (19.2) | 11 (13.8) | 26 (21.1) |  |
| 10-49% | 19 (19.0) | 136 (25.4) | 45 (41.3) |  | 15 (57.7) | 43 (53.8) | 52 (42.3) |  |
| < 10% | 74 (74.0) | 377 (70.5) | 56 (51.4) |  | 6 (23.1) | 26 (32.5) | 45 (36.6) |  |
| Neo-adjuvant chemotherapy | 6 (6.0) | 54 (10.1) | 32 (29.4) | <0.001 | 11 (42.3) | 42 (52.5) | 22 (17.7) | <0.001 |
| Breast surgery |  |  |  | 0.079 |  |  |  | 0.815 |
| Mastectomy | 69 (69.0) | 387 (72.3) | 89 (81.7) |  | 20 (76.9) | 66 (82.5) | 99 (80.5) |  |
| BCS | 31 (31.0) | 148 (27.7) | 20 (18.3) |  | 6 (23.1) | 14 (17.5) | 24 (19.5) |  |
| Axillary surgery |  |  |  | 0.626 |  |  |  | 0.055 |
| SLNB only | 27 (27.0) | 373 (69.9) | 81 (74.3) |  | 2 (7.7) | 13 (16.3) | 32 (26.0) |  |
| ALND | 73 (73.0) | 161 (30.1) | 28 (25.7) |  | 24 (92.3) | 67 (83.8) | 91 (74.0) |  |
| Adjuvant therapy | | | | | | | | |
| Chemotherapy | 43 (43.4) | 263 (49.3) | 90 (83.3) | <0.001 | 21 (80.8) | 58 (72.5) | 110 (90.2) | 0.005 |
| Endocrine therapy | 99 (100.0) | 534 (100.0) | 108 (100.0) | NA | 0 (0.0) | 0 (0.0) | 0 (0.0) | NA |
| Radiation therapy | 35 (35.4) | 212 (39.7) | 59 (54.6) | 0.007 | 16 (61.5) | 54 (67.5) | 61 (50.0) | 0.044 |

* Thirty patients received pathological complete response (pCR) after NAC.

Abbreviations: HER2, human epidermal growth factor receptor 2; CCI, Charlson Comorbidity Index; IDC, invasive ductal carcinoma; LVI, lymph vascular invasion; ER, estrogen receptor; PR, progestogen receptor; IHC, immunohistochemistry; sTIL, stromal tumor-infiltrating lymphocyte; ; BCS, breast conversing surgery; SLNB, sentinel lymph node biopsy; ALND, axillary lymph node dissection; NA, not available.

**Table S2. Univariate analysis of prognostic factors in whole population.**

| Characteristics | *P* value | |
| --- | --- | --- |
|  | OS | RFS |
| Age, year (< 55 vs ≥ 55) | 0.482 | 0.071 |
| Menstruation (Pre/peri vs post) | 0.538 | 0.304 |
| Cancer history (Yes vs No) | 0.501 | 0.449 |
| CCI (0-1 vs ≥ 2） | 0.624 | 0.230 |
| Histology (IDC vs non-IDC) | 0.079 | 0.760 |
| Grade (I-II vs III) | **<0.001** | **<0.001** |
| LVI (Yes vs No) | **<0.001** | **<0.001** |
| Tumor size, cm (≤ 2 vs > 2) | **<0.001** | **<0.001** |
| Lymph node status (Negative vs Positive) | **<0.001** | **<0.001** |
| ER (Positive vs Negative) | **<0.001** | **0.001** |
| PR (Positive vs Negative) | **<0.001** | **<0.001** |
| HER2 status | 0.563 | 0.888 |
| Ki67 (< 14% vs ≥ 14%) | **0.010** | 0.082 |
| sTILs per 10% increment | **0.008** | **0.001** |

Abbreviations: HER2, human epidermal growth factor receptor 2; OS, overall survival; RFS, recurrence-free survival; Pre/peri, Pre/perimenopausal; Post, postmenopausal; CCI, Charlson Comorbidity Index; IDC, invasive ductal carcinoma; LVI, lymph vascular invasion; ER, estrogen receptor; PR, progestogen receptor; IHC, immunohistochemistry; sTIL, stromal tumor-infiltrating lymphocyte.

**Table S3. Univariate analysis of prognostic factors in HER2-Low cohort.**

| Characteristics | *P* value | |
| --- | --- | --- |
|  | OS | RFS |
| Age, year (< 55 vs ≥ 55) | 0.651 | 0.109 |
| Menopause (Pre/peri vs post) | 0.723 | 0.159 |
| Cancer history (Yes vs No) | 0.502 | 0.411 |
| CCI (0-1 vs ≥ 2） | 0.549 | 0.088 |
| Histology (IDC vs non-IDC) | 0.079 | 0.284 |
| Grade (I-II vs III) | **<0.001** | **<0.001** |
| LVI (Yes vs No) | **<0.001** | **0.001** |
| Tumor size, cm (≤ 2 vs > 2) | **<0.001** | **<0.001** |
| Lymph node status (Negative vs Positive) | **<0.001** | **<0.001** |
| ER (Positive vs Negative) | **<0.001** | **<0.001** |
| PR (Positive vs Negative) | **<0.001** | **0.002** |
| Ki67 (< 14% vs ≥ 14%) | **0.002** | **0.025** |
| sTILs per 10% increment | 0.160 | 0.106 |

Abbreviations: HER2, human epidermal growth factor receptor 2; OS, overall survival; RFS, recurrence-free survival; Pre/peri, Pre/perimenopausal; Post, postmenopausal; CCI, Charlson Comorbidity Index; IDC, invasive ductal carcinoma; LVI, lymph vascular invasion; ER, estrogen receptor; PR, progestogen receptor; IHC, immunohistochemistry; sTIL, stromal tumor-infiltrating lymphocyte.

**Table S4. Multivariable Cox regression analysis of factors associated with prognosis in HER2-Low cohort.**

| Characteristics | OS | | |  | RFS | | |
| --- | --- | --- | --- | --- | --- | --- | --- |
|  | HR | 95%CI | *P* value |  | HR | 95%CI | *P* value |
| Grade |  |  | **0.029** |  |  |  | **0.003** |
| I-II | 0.50 | 0.21-0.98 |  |  | 0.52 | 0.34-0.80 |  |
| III | 1.00 |  |  |  | 1.00 |  |  |
| LVI |  |  | 0.511 |  |  |  | 0.285 |
| No | 0.75 | 0.32-1.58 |  |  | 0.65 | 0.30-1.43 |  |
| Yes | 1.00 |  |  |  | 1.00 |  |  |
| Tumor size, cm |  |  | **0.029** |  |  |  | **0.030** |
| ≤ 2 | 0.56 | 0.29-0.97 |  |  | 0.62 | 0.41-0.96 |  |
| > 2 | 1.00 |  |  |  | 1.00 |  |  |
| Lymph node status |  |  | **0.020** |  |  |  | **<0.001** |
| Negative | 0.59 | 0.27-0.90 |  |  | 0.46 | 0.30-0.70 |  |
| Positive | 1.00 |  |  |  | 1.00 |  |  |
| ER |  |  | <**0.001** |  |  |  | <**0.001** |
| Negative | 5.01 | 2.98-11.67 |  |  | 2.85 | 1.60-5.07 |  |
| Positive | 1.00 |  |  |  | 1.00 |  |  |
| PR |  |  | 0.521 |  |  |  | 0.874 |
| Negative | 1.26 | 0.64-2.49 |  |  | 1.06 | 0.601-1.81 |  |
| Positive | 1.00 |  |  |  | 1.00 |  |  |
| Ki67 |  |  | 0.109 |  |  |  | 0.463 |
| < 14% | 0.58 | 0.30-1.13 |  |  | 0.83 | 0.51-1.34 |  |
| ≥ 14% | 1.00 |  |  |  | 1.00 |  |  |
| sTILs per 10% increment | 0.79 | 0.62-0.90 | **0.007** |  | 0.81 | 0.72-0.94 | **0.009** |

Abbreviations: HER2, human epidermal growth factor receptor 2; OS, overall survival; RFS, recurrence-free survival; HR, hazard ratio; CI, confidence interval; CCI, Charlson Comorbidity Index; LVI, lymph vascular invasion; ER, estrogen receptor; PR, progestogen receptor; sTIL, stromal tumor-infiltrating lymphocytes.

**Table S5. Univariate analysis of prognostic factors in ER- / HER2-Low cohort.**

| Characteristics | *P* value | |
| --- | --- | --- |
|  | OS | RFS |
| Age, year (< 55 vs ≥ 55) | 0.367 | 0.470 |
| Menopause (Pre/peri vs post) | 0.554 | 0.187 |
| Cancer history (Yes vs No) | 0.730 | 0.358 |
| CCI (0-1 vs ≥ 2） | 0.091 | 0.052 |
| Histology (IDC vs non-IDC) | 0.308 | 0.339 |
| Grade (I-II vs III) | 0.402 | 0.294 |
| LVI (Yes vs No) | **0.010** | **0.022** |
| Tumor size, cm (≤ 2 vs > 2) | 0.299 | 0.223 |
| Lymph node status (Negative vs Positive) | **0.008** | **0.006** |
| PR (Positive vs Negative) | 0.221 | 0.168 |
| Ki67 (< 14% vs ≥ 14%) | 0.557 | 0.535 |
| sTILs per 10% increment | **0.004** | **0.001** |

Abbreviations: ER, estrogen receptor; HER2, human epidermal growth factor receptor 2; OS, overall survival; RFS, recurrence-free survival; Pre/peri, Pre/perimenopausal; Post, postmenopausal; CCI, Charlson Comorbidity Index; IDC, invasive ductal carcinoma; LVI, lymph vascular invasion; PR, progestogen receptor; IHC, immunohistochemistry; sTIL, stromal tumor-infiltrating lymphocyte.

**Table S6. Multivariable Cox regression analysis of factors associated with prognosis in ER- / HER2-Low cohort.**

| Characteristics | OS | | |  | RFS | | |
| --- | --- | --- | --- | --- | --- | --- | --- |
|  | HR | 95% CI | *P* value |  | HR | 95% CI | *P* value |
| CCI |  |  | **0.009** |  |  |  | **0.014** |
| 0-1 | 6.66 | 1.77-17.58 |  |  | 5.48 | 1.61-15.64 |  |
| ≥ 2 | 1.00 |  |  |  | 1.00 |  |  |
| LVI |  |  | **0.008** |  |  |  | **0.031** |
| No | 0.20 | 0.09-0.67 |  |  | 0.32 | 0.11-0.85 |  |
| Yes | 1.00 |  |  |  | 1.00 |  |  |
| Lymph node status |  |  | **0.038** |  |  |  | **0.027** |
| Negative | 0.30 | 0.10-0.94 |  |  | 0.28 | 0.09-0.87 |  |
| Positive | 1.00 |  |  |  | 1.00 |  |  |
| sTILs per 10% increment | 0.67 | 0.50-0.92 | **0.006** |  | 0.69 | 0.52-0.94 | **0.010** |

Abbreviations: ER, estrogen receptor; HER2, human epidermal growth factor receptor 2; OS, overall survival; RFS, recurrence-free survival; HR, hazard ratio; CI, confidence interval; CCI, Charlson Comorbidity Index; LVI, lymph vascular invasion; sTIL, stromal tumor-infiltrating lymphocytes.

**Table S7. Baseline characteristics of included and excluded patients from Jan. 2009 to Dec. 2013.**

| Characteristics | Included  N=943 (%) | Excluded  N=2014 (%) | *P* value* |
| --- | --- | --- | --- |
| Age, years (median, range) | 55.0 (23-92) | 54 (23-93) |  |
| Age, years |  |  | 0.221 |
| < 55 | 464 (49.2) | 1040 (51.6) |  |
| ≥ 55 | 479 (50.8) | 974 (48.4) |  |
| Menstruation |  |  | 0.038 |
| Pre/perimenopausal | 374 (39.7) | 881 (43.7) |  |
| Postmenopausal | 569 (60.3) | 1133 (56.3) |  |
| Prior cancer history |  |  | 0.919 |
| Yes | 37 (3.9) | 78 (3.9) |  |
| No | 905 (96.1) | 1936 (96.1) |  |
| CCI |  |  | 0.838 |
| 0-1 | 652 (69.1) | 1400 (69.5) |  |
| ≥ 2 | 291 (30.9) | 614 (30.5) |  |
| Histology |  |  | 0.387 |
| IDC | 849 (90.0) | 1792 (89.0) |  |
| non-IDC | 94 (10.0) | 222 (11.0) |  |
| Grade |  |  | 0.357 |
| I-II | 495 (52.5) | 932 (46.3) |  |
| III | 344 (36.5) | 597 (29.6) |  |
| Unknown | 104 (11.0) | 485 (24.1) |  |
| Tumor size, cm |  |  | 0.165 |
| ≤ 2 | 545 (57.8) | 1032 (51.2) |  |
| > 2 | 398 (42.2) | 843 (41.9) |  |
| Unknown | 0 (0.0) | 139 (6.9) |  |
| Lymph node status |  |  | 0.313 |
| Negative | 668 (70.8) | 1308 (64.9) |  |
| Positive | 275 (29.2) | 588 (29.2) |  |
| Unknown | 0 (0.0) | 138 (6.9) |  |
| LVI |  |  | 0.938 |
| Yes | 30 (3.2) | 63 (3.1) |  |
| No | 913 (96.8) | 1951 (96.9) |  |
| ER status |  |  | 0.116 |
| Positive | 734 (77.8) | 1444 (71.7) |  |
| Negative | 209 (22.2) | 477 (23.7) |  |
| Unknown | 0 (0.0) | 93 (4.6) |  |
| PR status |  |  | 0.144 |
| Positive | 548 (58.1) | 1061 (52.7) |  |
| Negative | 395 (41.9) | 860 (422.7) |  |
| Unknown | 0 (0.0) | 93 (4.6) |  |
| HER2 status |  |  | 0.054 |
| HER2-0 | 120 (12.7) | 309 (15.3) |  |
| HER2-Low | 600 (63.6) | 1161 (60.5) |  |
| HER2+ | 223 (23.6) | 449 (22.3) |  |
| Unknown | 0 (0.0) | 95 (4.7) |  |
| Ki67 |  |  | 0.001 |
| < 14% | 382 (40.5) | 899 (46.8) |  |
| ≥ 14% | 561 (59.5) | 1020 (53.2) |  |
| Unknown | 0 (0.0) | 95 (4.7) |  |
| Neo-adjuvant chemotherapy | 137 (14.5) | 260 (12.7) | 0.163 |
| Breast surgery |  |  | 0.585 |
| Mastectomy | 703 (74.5) | 1501 (74.5) |  |
| BCS | 240 (25.5) | 511 (25.4) |  |
| Unknown | 0 (0.0) | 2 (0.1) |  |
| Axillary surgery |  |  | 0.060 |
| SLNB only | 263 (28.0) | 475 (23.6) |  |
| ALND | 675 (72.0) | 1457 (72.3) |  |
| Unknown | 0 (0.0) | 82 (4.1) |  |
| Adjuvant therapy | N=939 | N=1995 |  |
| Chemotherapy | 570 (60.7) | 1155 (57.9) | 0.149 |
| Endocrine therapy | 721 (76.8) | 1550 (70.7) | 0.582 |
| Radiation therapy | 415 (44.2) | 783 (39.2) | 0.011 |

* Patients with unknown were not included in statistical analysis.

Abbreviations: HER2, human epidermal growth factor receptor 2; CCI, Charlson Comorbidity Index; IDC, invasive ductal carcinoma; LVI, lymph vascular invasion; ER, estrogen receptor; PR, progestogen receptor; IHC, immunohistochemistry; sTIL, stromal tumor-infiltrating lymphocyte; BCS, breast conversing surgery; SLNB, sentinel lymph node biopsy; ALND, axillary lymph node dissection.

**Table S8. Estimated 5-year and 8-year OS and RFS in patients stratified by ER and HER2 status.**

|  |  | OS (%) | | RFS (%) | |
| --- | --- | --- | --- | --- | --- |
|  |  | 5-year | 8-year | 5-year | 8-year |
| ER+ | HER2-0 | 91.9 | 90.1 | 86.9 | 83.1 |
|  | HER2-Low | 95.6 | 92.2 | 90.1 | 84.2 |
|  | HER2+ | 93.3 | 90.9 | 87.6 | 84.7 |
| ER- | HER2-0 | 76.2 | 71.4 | 85.7 | 80.4 |
|  | HER2-Low | 71.8 | 70.4 | 69.0 | 66.1 |
|  | HER2+ | 88.9 | 88.9 | 82.9 | 78.5 |

Abbreviations: OS, overall survival; RFS, recurrence-free survival; ER, estrogen receptor; HER2, human epidermal growth factor receptor 2.

**Table S9. Estimated 5-year and 8-year OS and RFS in patients in HER2-Low patients with different sTILs level and stratified by ER status.**

|  |  | OS (%) | | RFS (%) | |
| --- | --- | --- | --- | --- | --- |
|  |  | 5-year | 8-year | 5-year | 8-year |
| HER2-Low | <10% | 93.1 | 89.7 | 88.7 | 82.6 |
|  | 10-49% | 92.4 | 89.4 | 84.7 | 80.4 |
|  | ≥50% | 92.1 | 92.1 | 89.5 | 89.5 |
| ER+/HER2-Low | <10% | 95.1 | 91.8 | 90.8 | 84.6 |
|  | 10-49% | 97.0 | 93.2 | 87.9 | 82.3 |
|  | ≥50% | 96.0 | 96.0 | 92.0 | 92.0 |
| ER-/HER2-Low | <10% | 55.0 | 43.8 | 50.0 | 45.0 |
|  | 10-49% | 76.3 | 76.3 | 73.7 | 73.7 |
|  | ≥50% | 84.6 | 84.6 | 84.6 | 84.6 |

Abbreviations: OS, overall survival; RFS, recurrence-free survival; sTIL, stromal tumor-infiltrating lymphocytes; HER2, human epidermal growth factor receptor 2; ER, estrogen receptor.

**Table S10. sTILs according to ER and HER2 status in patients receiving NAC.**

|  | ER (%) | | *P* value | HER2 (%) | | | *P* value | ER+ (%) | | | *P* value | ER- (%) | | | *P* value |
| --- | --- | --- | --- | --- | --- | --- | --- | --- | --- | --- | --- | --- | --- | --- | --- |
|  | Positive | Negative |  | HER2-0 | HER2-Low | HER2+ |  | HER2-0 | HER2-Low | HER2+ |  | HER2-0 | HER2-Low | HER2+ |  |
| sTILs  (mean, range) | 9.0  (1-50) | 15.0  (1-70) | 0.024 | 16.2 (1-50) | 10.5 (1-70) | 12.5  (1-60) | 0.169 | 20.8 (5-50) | 7.4  (1-30) | 9.6  (1-40) | 0.005 | 13.7 (1-40) | 14.4 (1-70) | 16.7  (1-60) | 0.570 |
| sTILs categories |  |  | 0.001 |  |  |  | 0.490 |  |  |  | 0.137 |  |  |  | 0.665 |
| ≥ 50% | 0 (0.0) | 3 (4.0) |  | 0 (0.0) | 1 (1.0) | 2 (3.7) |  | 0 (0.0) | 0 (0.0) | 0 (0.0) |  | 0 (0.0) | 1 (2.4) | 2 (9.1) |  |
| 10-49% | 31 (33.7) | 43  (57.3) |  | 10 (58.8) | 40 (41.7) | 24 (44.4) |  | 4 (66.7) | 15 (27.8) | 12 (37.5) |  | 6 (54.5) | 25 (59.5) | 12 (54.5) |  |
| < 10% | 61 (66.3) | 29  (38.7) |  | 7 (41.2) | 55 (57.3) | 28 (51.9) |  | 2 (33.3) | 39 (72.2) | 20 (62.5) |  | 5 (45.5) | 16 (38.1) | 8  (36.4) |  |

Abbreviations: sTILs, stromal tumor-infiltrating lymphocytes; ER, estrogen receptor; HER2, human epidermal growth factor receptor 2; NAC, neoadjuvant chemotherapy.
